# Immunological modifications following chemotherapy are associated with delayed recurrence of ovarian cancer

**DOI:** 10.3389/fimmu.2023.1204148

**Published:** 2023-06-26

**Authors:** Nicholas Adzibolosu, Ayesha B. Alvero, Rouba Ali-Fehmi, Radhika Gogoi, Logan Corey, Roslyn Tedja, Hussein Chehade, Vir Gogoi, Robert Morris, Matthew Anderson, Julie Vitko, Clarissa Lam, Douglas B. Craig, Sorin Draghici, Thomas Rutherford, Gil Mor

**Affiliations:** ^1^ C. S. Mott Center for Human Growth and Development, Department of Obstetrics and Gynecology, Wayne State University School of Medicine, Detroit, MI, United States; ^2^ Department of Physiology, Wayne State University School of Medicine, Detroit, MI, United States; ^3^ Karmanos Cancer Institute, Wayne State University School of Medicine, Detroit, MI, United States; ^4^ Center of Molecular Medicine and Genetics, Wayne State University School of Medicine, Detroit, MI, United States; ^5^ Department of Obstetrics and Gynecology, University of South Florida Morsani College of Medicine, Tampa, FL, United States; ^6^ Department of Pathology and Cell Biology, University of South Florida Morsani College of Medicine, Tampa, FL, United States; ^7^ Department of Gynecologic Oncology, Memorial Sloan Kettering Cancer Center, New York, NY, United States; ^8^ Department of Computer Science, Wayne State University College of Engineering, Detroit, MI, United States; ^9^ Department of Oncology, Wayne State University School of Medicine, Detroit, MI, United States; ^10^ Advaita Corporation, Ann Arbor, MI, United States; ^11^ Division of Information and Intelligent Systems, Directorate for Computer and Information Science and Engineering, National Science Foundation, Alexandria, VA, United States

**Keywords:** ovarian cancer, chemoresistance, cold tumors, hot tumors, immune response

## Abstract

**Introduction:**

Ovarian cancer recurs in most High Grade Serous Ovarian Cancer (HGSOC) patients, including initial responders, after standard of care. To improve patient survival, we need to identify and understand the factors contributing to early or late recurrence and therapeutically target these mechanisms. We hypothesized that in HGSOC, the response to chemotherapy is associated with a specific gene expression signature determined by the tumor microenvironment. In this study, we sought to determine the differences in gene expression and the tumor immune microenvironment between patients who show early recurrence (within 6 months) compared to those who show late recurrence following chemotherapy.

**Methods:**

Paired tumor samples were obtained before and after Carboplatin and Taxol chemotherapy from 24 patients with HGSOC. Bioinformatic transcriptomic analysis was performed on the tumor samples to determine the gene expression signature associated with differences in recurrence pattern. Gene Ontology and Pathway analysis was performed using AdvaitaBio’s iPathwayGuide software. Tumor immune cell fractions were imputed using CIBERSORTx. Results were compared between late recurrence and early recurrence patients, and between paired pre-chemotherapy and post-chemotherapy samples.

**Results:**

There was no statistically significant difference between early recurrence or late recurrence ovarian tumors pre-chemotherapy. However, chemotherapy induced significant immunological changes in tumors from late recurrence patients but had no impact on tumors from early recurrence patients. The key immunological change induced by chemotherapy in late recurrence patients was the reversal of pro-tumor immune signature.

**Discussion:**

We report for the first time, the association between immunological modifications in response to chemotherapy and the time of recurrence. Our findings provide novel opportunities to ultimately improve ovarian cancer patient survival.

## Introduction

Ovarian Cancer is the deadliest gynecological malignancy. The most common subtype is High-Grade Serous Ovarian Cancer (HGSOC) which is mostly diagnosed at advanced stages due to vagueness of symptoms at early stages (1). HGSOC recurs in 70-80% of patients, with recurrence presenting rapidly, within 6 months post standard of care, or belatedly, up to 5 to 10 years post treatment (2). Despite modern advancements in ovarian cancer management, patient outcome for HGSOC still has room for improvement, with 5-year survival rate for advanced-stage disease being just about 30% (3). Cancer recurrence is the major cause of mortality in HGSOC patients and thus, needs to be tackled. However, the mechanisms which could be harnessed to delay or prevent recurrence of ovarian cancer are not yet known or fully understood.

Several mechanisms have been identified as mediating resistance to standard chemotherapy, potentially contributing to recurrence of HGSOC (4–6). These mechanisms can be broadly considered to be mechanisms intrinsic to the cancer cells versus microenvironmental factors affecting the cancer cell (6). Cancer cell intrinsic mechanisms include dysregulation of drug influx or efflux transporters, enhanced DNA repair pathways, as well as increased resistance to apoptosis (5). Cancer cell extrinsic mechanisms that contribute to treatment resistance and potentially ovarian cancer recurrence are adaptations in the tumor microenvironment (6). The ovarian cancer microenvironment comprises of pro-tumor versus anti-tumor immune cells, stromal cells (7), adipose-rich tissues in the peritoneal cavity (e.g., the omentum) (8), as well as the extracellular matrix stroma with secreted immune factors (7). Understanding the interplay between these mechanisms is critical to developing effective strategies to prevent or significantly delay ovarian cancer recurrence.

Since publication of the seminal work by Zhang et al. 20 years ago (9) which clearly showed that presence of intra-tumoral T lymphocytes correlated with improved patient outcomes in late-stage ovarian cancer, several studies have underscored the importance of the immune microenvironment in ovarian cancer (10–15). However, recent clinical trials of immunotherapy in ovarian cancer have shown only modest response rates (16–18). Furthermore, the effect of platinum- and taxane-based chemotherapy on the ovarian tumor immune microenvironment, and whether this effect differs between good responders versus poor responders at the cellular and molecular level is not yet known or well understood.

In this study, we sought to understand what differentiates HGSOC patients who recur as early as within 6 months following treatment from those with delayed recurrence, with focus on their response to Carboplatin and Taxol chemotherapy. Using the tools of bioinformatics and advancements in transcriptomics research, we tested the hypothesis that effective chemotherapy in HGSOC does not only directly kill cancer cells but also activates the immune system. We report for the first time that an efficient Carboplatin and Taxol chemotherapy induces immunological changes in HGSOC patient tumors and promotes an anti-tumor immune response.

## Materials and methods

### Patient selection

Forty-two (42) patients with FIGO Stage III/IV High Grade Serous Ovarian Cancer (HGSOC) were initially enrolled in this study. Tumor sample collection was obtained with informed consent and approved by IRB in Wayne State University and University of South Florida. Solid tumor samples were obtained from patients before and after completion of 6 cycles of Carboplatin and Taxol combination chemotherapy (C/T). All the samples were obtained from metastatic sites. Cancer cells from ascites was not included in the study. Eighteen (18) patients who did not meet quality control criteria (described below) at the sample evaluation, RNA extraction or cDNA library preparation stages were excluded from the final analyses. The pre-C/T or post-C/T samples for these patients had insufficient tumor percentage during pathologist evaluation (7 patients), low RNA extraction yield (4 patients) or poor-quality cDNA libraries (7 patients). Thus, 24 patients with paired pre-C/T and post-C/T RNA-seq data constituted the final study cohort. All tumor samples were less than 5 years old at the time of processing and sequencing.

### Sample preparation and RNA extraction

Tumor sample RNA extraction and sequencing was performed by Tempus (Tempus Labs Inc., Chicago, IL) according to the Tempus|RS.v2 RNA Assay and Bioinformatics Pipeline. For this assay, specimens from FFPE tumor samples were first stained with Hematoxylin and Eosin (H&E) on a slide. The H&E-stained slides were examined by a board-certified pathologist to assess the nucleated cell and tumor content of the specimen. Only samples with at least 20% tumor content were processed further for RNA extraction and cDNA library preparation. The Chemagic 360 system (Perkin Elmer) was used to extract total nucleic acid from the tumor samples. TURBO DNase (Invitrogen) was used to treat the extracted nucleic acid to degrade DNA, leaving tissue RNA for further processing.

### cDNA library preparation and sequencing

RNA from each sample was treated with heat and magnesium to fragment the RNA into equivalent sized fragments. cDNA libraries were prepared in a strand-specific fashion utilizing the KAPA RNA HyperPrep Kit for Illumina. IDT unique dual indexed unique molecular identifier adapters were ligated to the cDNA, after which cDNA library amplification was performed. Amplified cDNA libraries were captured by hybridization onto an IDT xGen Exome Research Panel v2 probe set for an enrichment step. Following this, the KAPA HiFi HotStart ReadyMix and primers from the KAPA Library amplification kit were used for the amplification step. Finally, Sequencing of the hybridized cDNA libraries was performed using the Illumina NovaSeq 6000 System to a 2x76 read length and an average sequencing depth of 50 million total reads. Generated raw RNA-seq data was de-multiplexed using the BCL2FASTQ software v2.17 (RRID:SCR_015058).

### RNA-seq data pre-processing by tempus

Quality control evaluation of the raw RNA-seq data was performed using MultiQC v1.11 (RRID:SCR_014982) with adapter sequences trimmed off using Skewer v0.2.2 (RRID:SCR_001151). Trimmed RNA-seq data was pseudo-aligned to the Ensembl GRCh37 human reference and quantified using Kallisto v0.44 (RRID:SCR_016582). Gene level abundance and corresponding transcripts per kilobase million (TPM) values for 20,061 genes was provided for each sample by Tempus Labs and was used for our downstream analyses. The biomaRt package v2.52.0 (RRID:SCR_019214) was used to convert ENSEMBL gene IDs to official HGNC symbols, resulting in an expression matrix with 19,057 genes.

### Data analysis

Further bioinformatics analysis was performed in the R programming environment using RStudio v4.2.0 (RRID:SCR_000432). The edgeR package v3.38.4 (RRID:SCR_012802) was used to perform differential gene expression (DGE) analysis. The paired nature of the pre-C/T and post-C/T data was considered in setting up the generalized linear model used by edgeR for the DGE analysis. Genes with FDR-adjusted p-value < 0.05 and absolute log2-foldchange > 0.6 were considered differentially expressed. Volcano plots and heatmaps of DGE analyses results were plotted using the ggplot2 package v3.4.0 (RRID:SCR_014601) and ComplexHeatmap package v2.12.1 (RRID:SCR_017270), respectively. Principal Component Analysis was performed using the PCAtools package v2.8.0 and Venn Diagrams were drawn using the ggvenn package v0.1.9. Boxplots of uniquely upregulated or downregulated genes were plotted with ggplot2. Gene Ontology (GO) and KEGG Pathway analyses were performed using AdvaitaBio’s iPathwayGuide software. Significantly enriched GO biological processes were those with adjusted p-value < 0.05, using the smallest common denominator pruning method. Significantly impacted KEGG pathways were those with FDR-adjusted combined overrepresentation and pathway perturbation p-value < 0.05.

### Immune deconvolution of bulk RNA-seq data

To estimate immune cell fractions for our gene expression dataset, normalized bulk RNA-seq data (TPMs) from our patient cohort was used as mixture matrix together with the LM22 signature matrix provided by CIBERSORTx (RRID:SCR_016955), which contains signature gene expression profile of 22 different immune cell types. These were used to impute immune cell fractions using the CIBERSORTx web software in relative mode with B-mode batch correction, disabled quantile normalization, and 1000 permutations. Results from the immune cell fraction estimation were visualized using ggplot2 for the stacked barplots and the ggpubr v0.4.0 (RRID:SCR_021139) and rstatix v0.7.1 (RRID:SCR_021240) packages for illustrating statistical comparisons as boxplots annotated with FDR-adjusted p-values.

### Statistics

For unpaired comparisons, the Wilcoxon Rank Sum Test was performed while paired pre-C/T and post-C/T comparison of immune cell fractions was performed using the Wilcoxon Signed Rank Test. Obtained p-values were corrected for multiple comparison using the Benjamini-Hochberg FDR method. FDR-adjusted p-values < 0.05 were considered statistically significant at 5% alpha level.

### Data availability

Raw data generated from bulk RNA sequencing in this study is publicly available in Gene Expression Omnibus (GEO) (RRID:SCR_005012) at GSE227100. Normalized gene expression data (RPKM) used for validation of immune deconvolution results was obtained from **Supplementary Data** associated with the publication by Javellana et al. (19).

## Results

### Patient selection and sample preparation for transcriptomic analysis

The main objective of this study was to identify mechanisms and factors within the tumor microenvironment which underlie the differential recurrence patterns observed in patients with High Grade Serous Ovarian Cancer (HGSOC). To achieve this objective, we enrolled a cohort of 42 patients with HGSOC diagnosed at FIGO stages III/IV. All patients had initial tumor biopsies prior to beginning chemotherapy with 6 cycles of Carboplatin and Taxol (C/T). Afterwards, the patients underwent post-chemo surgery, which provided the post-C/T sample. All the samples were obtained from metastatic sites. Cancer cells from ascites was not included in the study. Thus, each patient had paired pre- and post-C/T tumor samples for transcriptomic analysis. Following debulking surgery, patients received additional adjuvant chemotherapy and were followed up for signs of remission, development of recurrent disease or any other complications.

**Figure 1:**
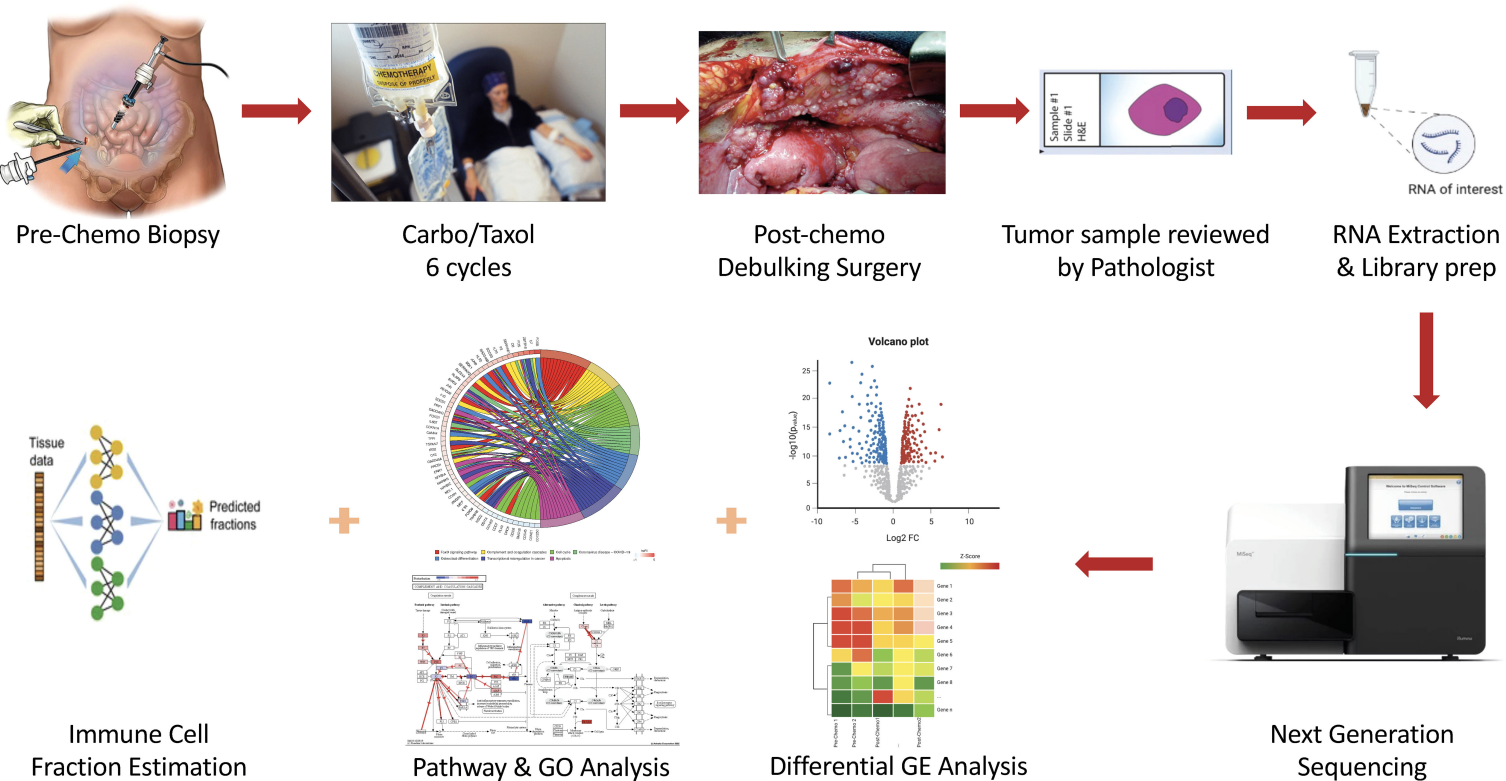
Schematic illustration of the experimental workflow followed in this study. Enrolled HGSOC patients had pre-chemo biopsy taken, after which they received 6 cycles of Carboplatin and Taxol (C/T). Following this, interval debulking surgery was performed and post-chemo samples obtained. Both pre-C/T and post-C/T samples were prepared and examined by a Board-Certified Pathologist, after which RNA extraction, cDNA library preparation and sequencing were performed on high quality samples. RNA-seq data was then analyzed in multiple steps for differential gene expression detection, pathway impact, and gene ontology enrichment detection as well as immune cell fraction estimation.

The tumor samples obtained were initially paraffin-fixed and formalin-embedded for long-term preservation. Tumor blocks were later sectioned and stained using Hematoxylin & Eosin dye for microscopy. Stained tumors were examined by a board-certified pathologist to ensure that only samples that had sufficient tumor purity (i.e., > 20%) were further processed. Tumors that met the above purity criteria were processed for RNA extraction, cDNA library preparation, and sequencing. Only samples with high quality cDNA libraries were sequenced and considered in downstream analyses. **Figure 1** summarizes the experimental workflow followed in this study.

Out of the 42 patients initially enrolled, only 24 patients (i.e., 24 pairs of pre- and post-C/T samples) met the inclusion criteria of sufficient tumor content and high-quality cDNA libraries. These constituted the final cohort for this study. The median age at diagnosis for these 24 patients was 63.5 years (Range = 47 – 74 years) (**Table 1**). Initial global visualization of the gene expression profiles of all 24 pairs of samples using Principal Component Analysis (PCA), t-Distributed Stochastic Neighbor Embedding (t-SNE) and Uniform Manifold Approximation and Projection (UMAP) showed that samples clustered based on time of sampling in relation to chemotherapy (i.e., whether pre-C/T or post-C/T) rather than time of future recurrence (**Supplementary Figures 1**–**3**).

**Table 1:** Summary of Patient Characteristics.

Characteristics	Value
Median age at diagnosis, years	63.5 (Range = 47 – 74)
FIGO Stage, n (%)	
IIIC	16 (66.67%)
IVA	4 (16.67%)
IVB	3 (12.5%)
X	1 (4.16%)
CA-125, U/mL	
Median CA-125 at diagnosis	1502 (Range = 41.6 - 43,665)
Median CA-125 before surgery	27.3 (Range = 9.6 - 445)
Tumor purity, % (standard deviation)	
Mean tumor purity pre-C/T	59.2 (SD = 19.5)
Mean tumor purity post-C/T	50.0 (SD = 18.9)
Late Recurrence patients (n = 13)	
Median age at diagnosis, years	65 (Range = 54 - 73)
Early Recurrence patients (n = 11)	
Median age at diagnosis, years	62 (Range = 47 - 74)

### Chemotherapy induces significant transcriptomic changes related to oxygen transport, cell cycle, and immunological pathways in ovarian cancer patients

**Figure 2:**
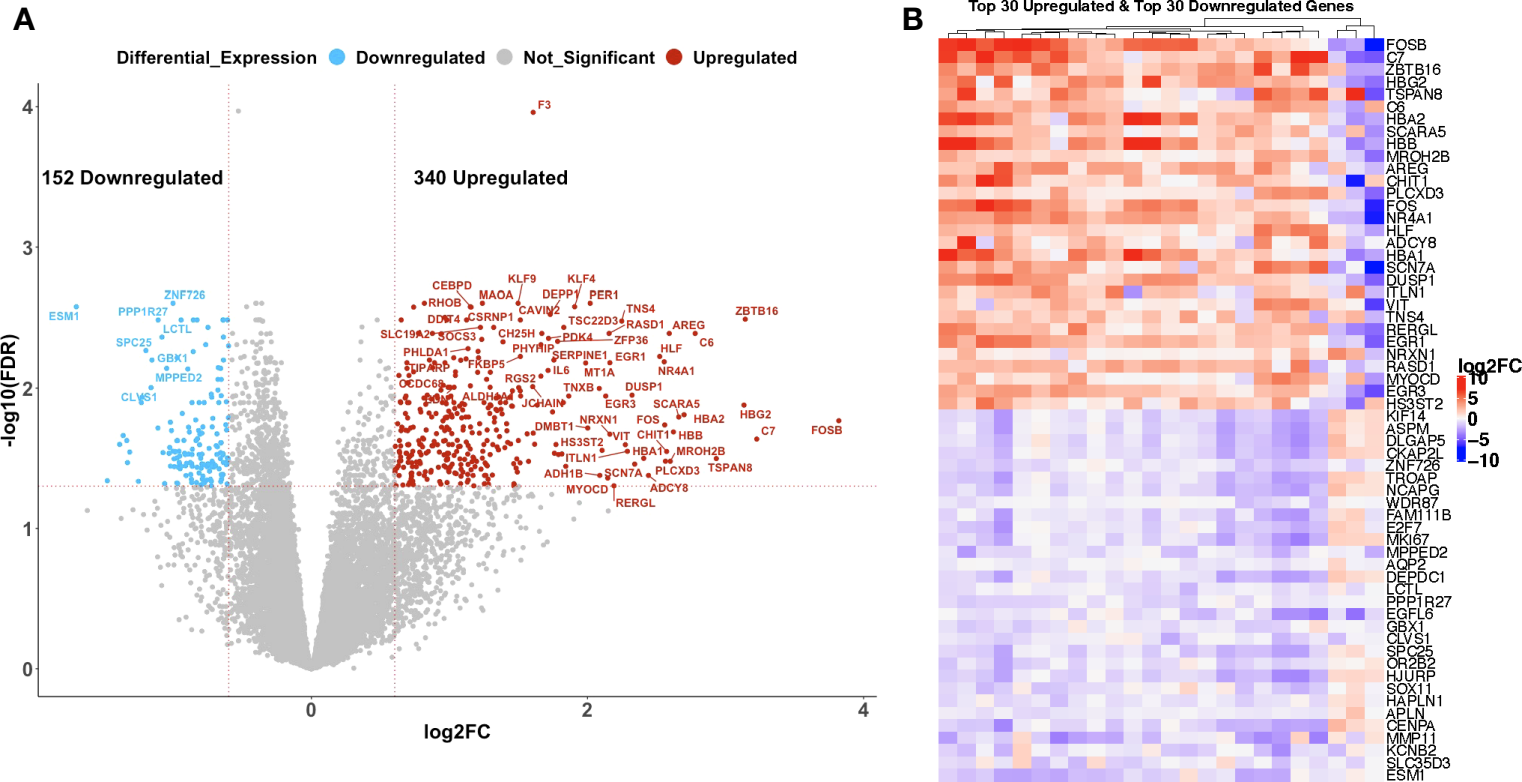
Chemotherapy induced gene expression changes in high grade serous ovarian cancer patients. **(A)** A volcano plot showing significantly upregulated (red) or downregulated (blue) genes (FDR < 0.05 and |log2-foldchange| > 0.6) post-C/T. Genes with FDR < 0.01 or |log2-foldchange| > 2 are labeled with HGNC symbols. **(B)** A heatmap showing the top 30 upregulated (red) and top 30 downregulated (blue) genes post-C/T (log2-foldchange was used as the ranking metric). For each gene, log2-foldchange in transcripts per kilobase million (TPM) values was computed per patient, comparing pre-C/T versus post-C/T expression values (pre-C/T as reference). Each column corresponds to a distinct patient.

To determine the effect of C/T on the gene expression profile of ovarian tumors, we compared paired pre- versus post-C/T RNA-sequence data for all 24 patients using the edgeR package in RStudio. With a Benjamini-Hochberg FDR-adjusted p-value threshold of 0.05 and an absolute log2-fold change (or log2FC) threshold of 0.6, we identified 340 upregulated and 152 downregulated genes post-C/T (**Figure 2A**). Coagulation factor III (also known as Tissue Factor or *F3*) – a marker of tissue damage – had the most significant FDR-adjusted p-value (FDR=1.096x10^-4^) and showed uniform upregulation post-C/T across all 24 patients (**Figure 2A**, **Supplementary Data 1**). The heatmap in **Figure 2B** shows the log2-fold change of the top 30 upregulated genes and top 30 downregulated genes for each patient. Among these differentially expressed genes (DEGs), the most highly upregulated gene post-C/T was *FOSB* (log2-fold change = 3.82) (**Figures 2A, B**, **Supplementary Data 1**), a member of the activator protein 1 (AP-1) family of genes. *AP-1* genes have been reported recently as being highly upregulated post-chemotherapy in HGSOC (19). Other *AP-1* genes including *c-FOS*, *c-JUN*, *JUNB*, and *JUND* were also significantly upregulated in our post-C/T samples (**Supplementary Data 1**) showing that our findings are consistent with current literature. Proteins encoded by *AP-1* genes function as dimeric transcription factors that are important for diverse cellular processes including cellular proliferation, apoptosis, and differentiation (20). We also found that among all DEGs, the pro-angiogenic endothelial cell-specific molecule 1 (*ESM1*) demonstrated the most remarkable downregulation following C/T (log2-fold change = -1.71) (**Figures 2A, B**, **Supplementary Data 1**). High expression of *ESM1* has been described in multiple cancers as an unfavorable prognostic marker (21–24). Therefore, its downregulation following C/T could be a desirable occurrence since it could also suggest decreased angiogenesis in ovarian tumors post-C/T. Thus, comparing our cohort of pre- and post-C/T samples, we observed the differential expression of genes associated with cell proliferation and cell death as well as angiogenesis. These results demonstrate that C/T was actively changing the tumor transcriptome of our HGSOC cohort.

**Figure 3:**
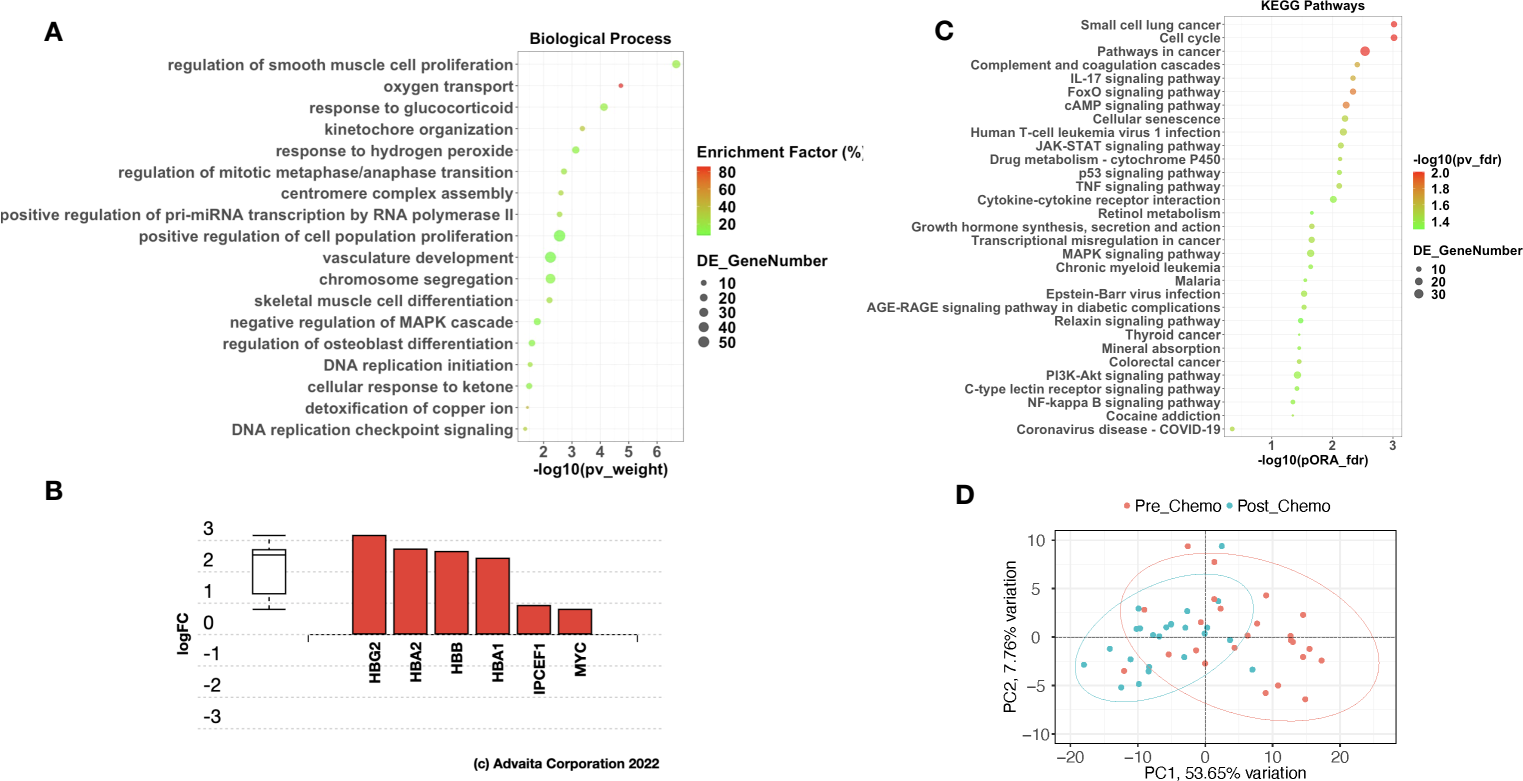
Chemotherapy impacted pathways related to cancer, cell cycle, and immune response in HGSOC patients. **(A)** A dot-plot showing the significantly enriched (adjusted p-value < 0.05) gene ontology (GO) biological processes identified with the iPathwayGuide software. pv_weight = adjusted p-values obtained using the Smallest Common Denominator pruning method for correcting for multiple comparisons. Enrichment Factor (%) = 100*(number of genes differentially expressed in a GO term)/(number of all genes on the GO term). Highest % enrichment is colored red with lowest enrichment colored green according to the color scale. DE_GeneNumber = number of differentially expressed genes in the GO term. **(B)** Differentially expressed genes in the Oxygen Transport GO term. Red color indicates gene upregulation. **(C)** A dot-plot showing the significantly impacted KEGG pathways identified with the iPathwayGuide software. pORA_fdr = FDR-adjusted p-values obtained from pathway overrepresentation analysis. pv_fdr = FDR-adjusted global p-values obtained from combining pORA and pathway perturbation p-values. The most significant global pv_fdr is colored red with the least significant global pv_fdr colored green according to the color scale. DE_GeneNumber = number of differentially expressed genes in the KEGG pathway term. **(D)** Principal Component Analysis using top 30 upregulated and top 30 downregulated genes shows partial separation of pre-C/T samples from post-C/T samples.

We next sought to understand the functional impact of all the observed DEGs post-C/T. Thus, we performed Gene Ontology (GO) and KEGG pathway analysis using AdvaitaBio’s iPathwayGuide software and identified significantly enriched biological processes and impacted pathways. Surprisingly, adjusting the p-values associated with the identified biological processes using the Benjamini-Hochberg FDR approach yielded 494 biological processes as being significantly enriched (**Supplementary Data 2**). We observed, however, that these GO terms contained redundancies (e.g., “response to stimulus” as well as “cellular response to stimulus” were both identified as distinct significantly enriched biological processes (**Supplementary Data 2**). This is an intrinsic challenge with standard Gene Ontology analysis, partly due to the parent-child hierarchical structure of GO terms (25). To exclude these redundancies and identify truly unique biological processes impacted by C/T, we utilized iPathwayGuide’s functionality of correcting for multiple comparison using the smallest common denominator pruning method (26, 27). This yielded 18 biological processes identified as significantly enriched following C/T (i.e., adjusted p-value < 0.05) (**Figure 3A**). The process with the highest enrichment factor (i.e., percentage of DEGs among all measured genes within the GO term being considered) was Oxygen Transport (**Figure 3A**). In this process, out of the 7 measured genes, 6 genes were upregulated post-C/T (**Figure 3B**). Four (4) out of the 6 upregulated genes – *HBG2*, *HBA2*, *HBB*, and *HBA1* – code for different subunits of the hemoglobin molecule (**Figure 3B**). The upregulation of oxygen transport post-C/T could be an adaptational response by the cancer to support tumor growth and overcome the effects of chemotherapy. This is reasonable because, for instance, we previously observed the pro-angiogenic gene *ESM1* to be downregulated by chemotherapy (**Figures 2A, B**), and Taxol is known to have anti-angiogenic activity (28). In addition to the principal role of hemoglobin as the main oxygen career in humans, and indeed in all mammals, some hemoglobin subunits have been suggested to be important in the modulation of cancer progression (29–31). Therefore, the upregulation of these hemoglobin subunit genes following chemotherapy is worthy of future investigation.

Pathway analysis for the DEGs showed 31 KEGG pathways impacted post-C/T (**Figure 3C**). The most significantly impacted pathways in our cohort were related to cancer, cell cycle and immunological pathways (**Figure 3C**). The immunological pathways include IL-17 signaling pathway, JAK-STAT signaling, and TNF signaling, among several others (**Figure 3C**). These findings suggest that modification of immunological pathways occurs in HGSOC patients following C/T treatment.

Finally, we performed Principal Component Analysis using the top 30 upregulated genes and the top 30 downregulated genes shown in **Figure 2B** (i.e., 60 genes in total) to determine if these DEGs alone could distinguish post-C/T samples from pre-C/T samples. As seen in **Figure 3D**, the Principal Component 1 (PC1) on the x-axis computed from these 60 genes contributed to more than 50% (53.65%) of the variation within the data. However, despite some separation, the 95% confidence interval ellipses drawn around the pre- versus post-C/T samples still showed some degree of overlap (**Figure 3D**). It is possible that this overlap could be due to some patients who showed poor outcomes after treatment and without significant DEGs between their pre- and post-C/T samples. This led us to hypothesize that HGSOC patients show differential changes in gene expression following chemotherapy, depending on each patient’s recurrence pattern following treatment. The results we obtained from testing this hypothesis are described in the following sections.

### Ovarian cancer patients with delayed recurrence, but not those with early recurrence, demonstrate significant gene expression changes following chemotherapy

To determine if patients who show better clinical response to C/T have a unique gene expression profile compared to patients with poorer response, we classified the 24 patients based on their recurrence pattern. Eleven (11) out of the 24 patients presented with disease within 6 months post-C/T and constituted the “Early Recurrence” class. Thirteen (13) out of the 24 patients did not show recurrent disease within 6 months and constituted the “Late Recurrence” class. The median age at diagnosis for patients within each recurrence class were comparable: 65 years (Range = 54 – 73 years) for “Late Recurrence” and 62 years (Range = 47 – 74 years) for “Early Recurrence” (*P* = 0.8; Two-Sample Student’s t-test). For each recurrence class, we performed paired pre- versus post-C/T comparisons to identify significant DEGs. Our goal was to determine if the two recurrence patterns were associated with a specific transcriptomic profile.

**Figure 4:**
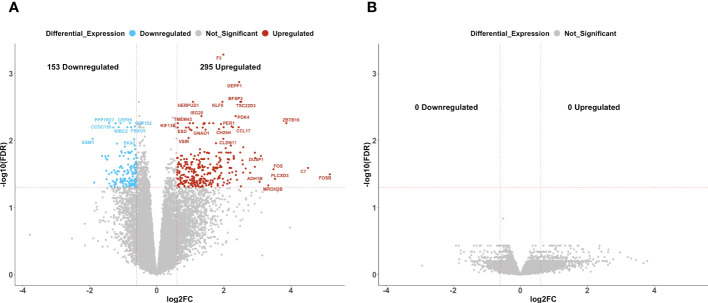
Ovarian cancer patients with delayed recurrence, but not those with early recurrence, demonstrated significant gene expression changes post-C/T. **(A)** A volcano plot showing significantly upregulated (red) or downregulated (blue) genes (FDR < 0.05 and |log2-foldchange| > 0.6) post-C/T in Late Recurrence patients. Genes with FDR < 0.01 or |log2-foldchange| > 3 are labeled with HGNC symbols. **(B)** A volcano plot showing that there was no significantly upregulated or downregulated gene (FDR < 0.05 and |log2-foldchange| > 0.6) post-C/T in Early Recurrence patients.

**Figure 5:**
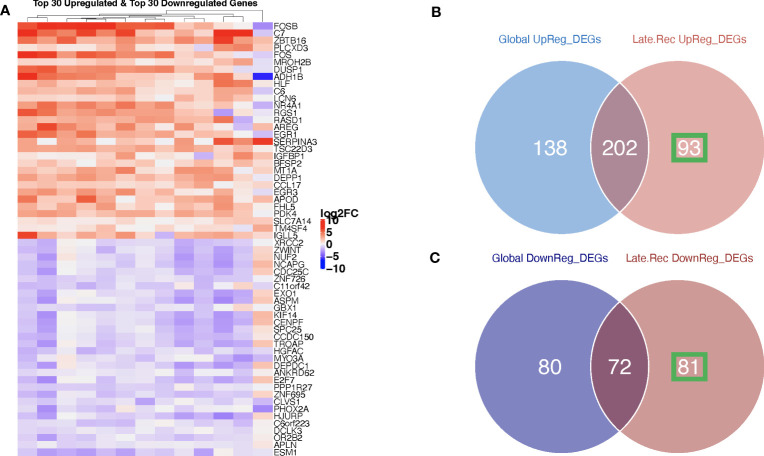
Ovarian cancer patients with delayed recurrence showed uniquely upregulated and uniquely downregulated genes post-C/T. **(A)** A heatmap showing the top 30 upregulated (red) and top 30 downregulated (blue) genes post-C/T in Late Recurrence patients (log2-foldchange was used as the ranking metric). For each gene, log2-foldchange in transcripts per kilobase million (TPM) values was computed per patient, comparing pre-C/T versus post-C/T expression values (pre-C/T as reference). Each column corresponds to a distinct patient. **(B)** A Venn diagram comparing upregulated genes from the initial Global differential gene expression analysis with upregulated genes from the Late Recurrence differential gene expression analysis. 93 genes (in green square) were uniquely upregulated in Late Recurrence patients post-C/T. **(C)** A Venn diagram comparing downregulated genes from the initial Global differential gene expression analysis with downregulated genes from the Late Recurrence differential gene expression analysis. 81 genes (in green square) were uniquely downregulated in Late Recurrence patients post-C/T.

In patients with delayed recurrence, we observed significant upregulation of 295 genes and significant downregulation of 153 genes in post-C/T samples compared to pre-C/T samples (**Figure 4A**, **Supplementary Data 3**). Interestingly, in patients with early recurrence, no significant DEGs were observed when post-C/T samples were compared to their corresponding pre-C/T samples (**Figure 4B**, **Supplementary Data 4**). Twelve out of the thirteen patients in the Late Recurrence group showed uniformly positive and negative log2-fold changes for the top 30 most upregulated and the top 30 most downregulated genes identified, respectively (**Figure 5A**). This uniformity in gene expression change further suggested to us a potential association between delayed recurrence of disease and the observed gene expression signatures. The top 30 upregulated and top 30 downregulated DEGs for the late recurrence class shown in **Figure 5A** shared major similarities with the preceding global analysis (**Figure 2B**). Therefore, to determine if the gene expression changes that we observed from the initial global pre- versus post-C/T analysis (which had ignored recurrence sub-classification, **Supplementary Data 1**) were driven by the Late Recurrence group (**Supplementary Data 3**), we compared the lists of all DEGs from the two analyses.

Nearly 70% (202 out of 295) of the upregulated genes in patients with delayed recurrence were also found to be upregulated in the initial global comparison (**Figure 5B**; **Supplementary Data 5**). Likewise, 72 out of the 153 downregulated genes in the Late Recurrence group were also downregulated in the initial global comparison (**Figure 5C**; **Supplementary Data 6**). These results suggest that most of the initial global differential gene expression profile observed were driven by the post-C/T gene expression changes occurring in the Late Recurrence patient sub-cohort; especially since the corresponding log2-foldchanges were greatest in magnitude in the Late Recurrence group (**Supplementary Datas 5, 6**). When considered alone, patients with early recurrence did not show any significant DEGs post-C/T (**Figure 4B**).

Interestingly, 93 upregulated genes and 81 downregulated genes were significantly differentially expressed in the Late Recurrence group but lost their significant differential expression in the global analyses (**Figures 5B, C**, respectively). Since the global analyses included patients who showed early recurrence, we believe that these 93 and 81 genes are genes whose differential expression post-C/T is unique to patients with late recurrence.

**Figure 6:**
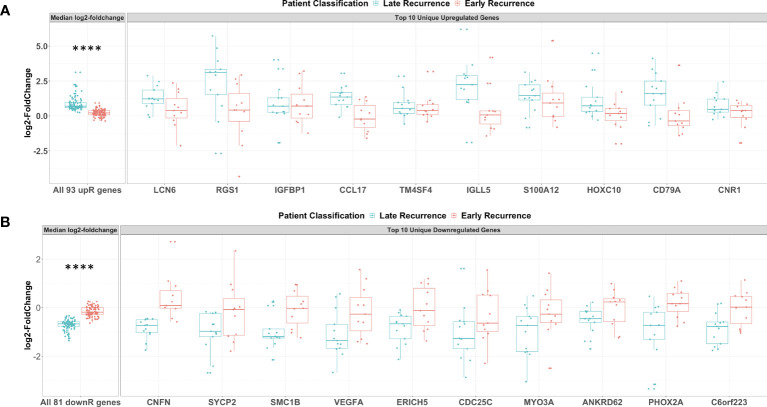
Genes uniquely upregulated post-C/T in Late Recurrence patients were associated with immunological response while uniquely downregulated genes involved cell cycle, DNA repair and angiogenesis. **(A)** Boxplots of all 93 uniquely upregulated genes (left facet) and top 10 uniquely upregulated genes (right facet). Log2-foldchanges post-C/T are shown separately for Late Recurrence (blue) and Early Recurrence (red) patients. For left facet (i.e., all 93 genes), the median log2-foldchange per gene was plotted as an individual dot. For right facet (i.e., top 10 genes), the individual log2-foldchange per patient was plotted as an individual dot. The p-value shown was obtained from Wilcoxon Rank Sum Test. **(B)** Boxplots of all 81 uniquely downregulated genes (left facet) and top 10 uniquely downregulated genes (right facet). Log2-foldchanges post-C/T are shown separately for Late Recurrence (blue) and Early Recurrence (red) patients. For left facet (i.e., all 81 genes), the median log2-foldchange per gene was plotted as an individual dot. For right facet (i.e., top 10 genes), the individual log2-foldchange per patient was plotted as an individual dot. The p-value shown was obtained from Wilcoxon Rank Sum Test. ****P < 0.0001.

To confirm that these unique DEGs were not markedly changed by C/T in those patients who showed early recurrence, we extracted the corresponding log2-fold changes per patient for all 93 upregulated (**Figure 6A**) and all 81 downregulated genes (**Figure 6B**). Together, all the 93 genes uniquely upregulated post-C/T in the Late Recurrence group showed significantly more positive log2-foldchange post-C/T in Late Recurrence patients compared to Early Recurrence patients (*P* = 2.77e-26) (**Figure 6A**). Similarly, all the 81 genes uniquely downregulated post-C/T in the Late Recurrence group showed significantly more negative log2-foldchange post-C/T in Late Recurrence patients compared to Early Recurrence patients (*P* = 4.33e-26) (**Figure 6B**).

For patients in the Late Recurrence class, we observed that most of the top 10 uniquely upregulated genes shown in **Figure 6A** were increased by a greater magnitude of log2-foldchange post-C/T whereas these genes showed median log2-foldchanges barely different from zero (0) in Early Recurrence patients (**Figure 6A**). Likewise, while all the top 10 uniquely downregulated genes shown demonstrated greater magnitude of decrease post-C/T in Late Recurrence patients, the corresponding log2-foldchange values for the Early Recurrence group were barely different from zero (0) (**Figure 6B**).

Among the top 10 uniquely upregulated genes, we identified some genes that we believe could have contributed to the different recurrence classes. Interestingly, these comprised mostly of immune-related genes. Late Recurrence patients, but not Early Recurrence patients, showed significant upregulation of LCN6, RGS1, CCL17, IGLL5, and CD79A (**Figure 6A**). Scientific literature is currently limited on the biology of Lipocalin 6 (LCN6), but it was recently identified as one of a panel of nine immune-related genes able to predict overall survival class of epithelial ovarian cancer patients as “low-risk” or “high-risk” (32). The Regulator of G-protein Signaling protein 1 (RGS1) is known to regulate chemokine-induced lymphocyte migration (33), and has been described as a marker of human CD69+ tissue-resident memory T cells (34). High intra-tumoral expression of the C-C motif Chemokine Ligand 17 (CCL17) has been found to promote T cell infiltration of tumors thereby contributing to improved survival in some cancers (35). However, CCL17 has also been found to facilitate trafficking of CCR4-expressing regulatory T cells into some tumors thereby mediating resistance to immunotherapy in these cases (36). Therefore, the role of CCL17 in the ovarian tumor microenvironment is worthy of further investigation. The biological function of the Immunoglobulin Lambda-Like polypeptide 5 (IGLL5) is yet to be described comprehensively but IGLL5 levels were recently found to be positively correlated with tumor-infiltrating immune cells in renal cancer (37). Lastly, *CD79A* codes for the alpha chain of a B cell antigen receptor complex-associated protein which is required for B cell development. CD79A is important for B cell activation (38) as well as surface IgM expression (39) following antigen - B cell receptor complex formation. Its significant upregulation following C/T in patients with delayed recurrence could suggest activation of B cells by C/T.

Among the top 10 uniquely downregulated genes, we once more identified some interesting genes that we believe could have contributed to the different recurrence classes. Late Recurrence patients, but not Early Recurrence patients, showed significant downregulation of SYCP2, SMC1B, VEGFA, and CDC25c (**Figure 6B**). Synaptonemal Complex Protein-2 (SYCP2) is the largest protein component of the synaptonemal complex which is required for homologous recombination of chromosomes and plays critical roles during double-strand DNA break repair in somatic cells (40, 41). Structural Maintenance of Chromosomes protein-1B (SMC1B) is an important component of cohesin which holds sister chromatids together and facilitates double-strand DNA break repair via the homologous recombination pathway (42). Therefore, downregulation of SYCP2 and SMC1B post-C/T in Late Recurrence patients suggests C/T-induced impairment of DNA repair mechanisms, potentially explaining the better treatment outcome. In addition, significant downregulation of the pro-angiogenic Vascular Endothelial Growth Factor A (VEGFA) post-C/T in Late Recurrence patients suggests that, in these patients, C/T could counteract the pro-tumor effects often mediated by VEGFA. This could contribute to the delayed recurrence in this group of patients compared to those with Early Recurrence. Cell Division Cycle 25c (CDC25C) is the critical phosphatase which removes an inhibitory phosphate on the cyclin-dependent kinase CDK1 thereby transitioning dividing cells from G2 phase into M phase. The significant downregulation of CDC25C post-C/T in Late Recurrence patients but not in Early Recurrence patients suggests that HGSOC patients in whom C/T induces cell cycle arrest are more likely to experience delayed recurrence of cancer, as one would expect.

Interestingly, comparing pre-C/T RNA-seq data in Late Recurrence versus Early Recurrence groups yielded no statistically significant differences in gene expression (**Supplementary Data 7**). This suggests that the gene expression changes in response to C/T, rather than a predefined pre-C/T transcriptomic signature alone, was associated with the observed recurrence pattern.

In summary, our results show that HGSOC patients who recur later than 6 months post standard of care have significant gene expression changes following C/T while those who recur within 6 months after treatment have no significant gene expression changes following C/T. And that differential expression of immune-related genes could be associated with delayed recurrence of ovarian cancer.

### Ovarian cancer patients with delayed recurrence after treatment showed upregulation of complement pathway and positive regulation of T cell differentiation following chemotherapy

**Figure 7:**
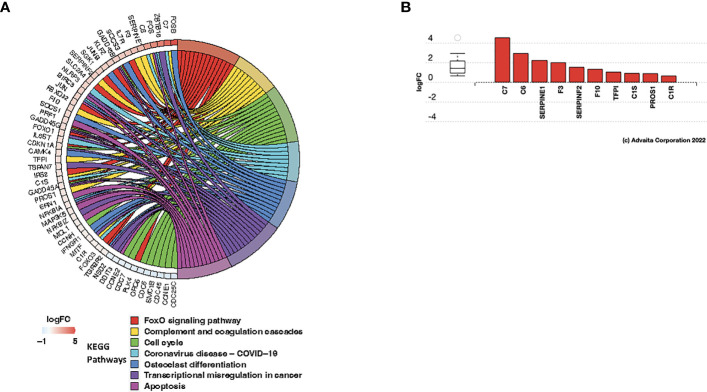
C/T induced immunological modifications in late recurrence ovarian cancer patients. **(A)** Circos plot of significantly impacted KEGG pathways in Late Recurrence patients identified using the iPathwayGuide software. Significantly impacted pathways were those with FDR-adjusted global p-values (i.e., combined pORA and pPerturbation) < 0.05. Pathways are color-coded and shown in the right half of the plot (legend shown beneath the plot). All differentially expressed genes within the impacted pathways are shown in the left half of the plot along with their corresponding log2-foldchanges shown in adjacent boxes, color-coded according to the logFC color scale beneath the plot. Negative logFC (blue) indicates downregulation and positive logFC (red) indicates upregulation. **(B)** Differentially expressed genes in the Complement and Coagulation Cascade Pathway. Red color indicates gene upregulation.

We next determined the functional significance of the post-C/T differential gene expression that we observed to be unique to patients in the Late Recurrence group. We performed pathway impact and gene ontology enrichment analyses using AdvaitaBio’s iPathwayGuide software. At a significance level of 5% using FDR-adjusted p-values, we found 7 KEGG pathways to be significantly impacted post-C/T in the Late Recurrence patient sub-cohort (**Figure 7A**). This comprised of FoxO Signaling Pathway, Complement and Coagulation Cascades, Cell Cycle, Coronavirus Disease – COVID-19, Osteoclast Differentiation, Transcriptional Misregulation in Cancer, and Apoptosis (**Figure 7A**). Most of the genes within the Cell Cycle pathway were downregulated while genes within the Apoptotic pathway were upregulated (**Figure 7A**), as one would expect. Genes within the Complement and Coagulation Cascade were also significantly upregulated (**Figures 7A, B**). Interestingly, there was significant upregulation of complement components C7 and C6 which form parts of the Membrane Attack Complex (**Figure 7B**, **Supplementary Figure 4**) that inserts into the membranes of cells (e.g., cancer cells) during complement-mediated cell death. Furthermore, complement components C1R and C1S which are the catalytic subunits of the C1 complex that activates Complement Cascade via the Classical Pathway (43) were also upregulated (**Figure 7B**, **Supplementary Figure 4**). The tissue damage marker, Tissue Factor F3, an activator of the extrinsic arm of the Coagulation Cascade was also found to be upregulated post-C/T in the group of patients with delayed recurrence (**Figure 7B**, **Supplementary Figure 4**). Taken together, these results demonstrate that a critical finding in patients in the Late Recurrence group is that chemotherapy significantly promoted tumor tissue damage associated with an activation of immune responses such as antigen-antibody complex-mediated complement activation.

**Figure 8:**
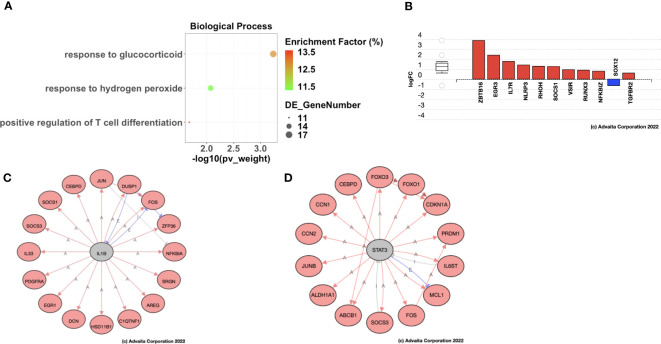
Ovarian cancer patients with delayed recurrence showed enrichment of positive regulation of T cell differentiation post-C/T. **(A)** A dot-plot showing the significantly enriched (adjusted p-value < 0.05) gene ontology (GO) biological processes identified with the iPathwayGuide software. pv_weight = adjusted p-values obtained using the Smallest Common Denominator pruning method for correcting for multiple comparisons. Enrichment Factor (%) = 100*(number of genes differentially expressed in a GO term)/(number of all genes on the GO term). Highest % enrichment is colored red with lowest % enrichment colored green according to the color scale shown. DE_GeneNumber = number of differentially expressed genes in the GO term. **(B)** Differentially expressed genes in the Positive Regulation of T cell Differentiation GO term. Red color indicates gene upregulation and blue color indicates gene downregulation. **(C)** STRING network diagram of IL1B as an upstream regulator predicted as activated due to the shown IL1B targets being upregulated (red fill). Red arrows indicate activation, blue lines with perpendicular blocked ends indicate expression, and grey lines with perpendicular blocked ends indicate inhibition. **(D)** STRING network diagram of STAT3 as an upstream regulator predicted as activated due to the shown STAT3 targets being upregulated (red fill). Red arrows indicate activation, blue lines with perpendicular blocked ends indicate expression, and grey lines with perpendicular blocked ends indicate inhibition.

To further characterize the molecular mechanisms underlying the different recurrence patterns observed in HGSOC patients, we performed Gene Ontology analysis to identify significantly enriched biological processes associated with Late Recurrence. Using a Benjamini-Hochberg FDR-adjusted p-value < 0.05 as significance criterion, we identified 340 biological processes that were significantly enriched, most of which were immunological (**Supplementary Data 8**). Once more, the smallest common denominator pruning of these GO terms yielded 3 key biological processes that were significantly enriched post-C/T. These were Response to Lipid, Response to Hydrogen Peroxide, and Positive Regulation of T Cell Differentiation (**Figure 8A**). Of these 3 enriched biological processes, the process with the highest enrichment factor was Positive Regulation of T Cell Differentiation. It was interesting to find that the most DEGs within the Positive Regulation of T Cell Differentiation process included upregulation of *ZBTB16* and *EGR3*, among several others (**Figure 8B**). *ZBTB16* encodes the transcription factor PLZF which drives the expansion and differentiation of populations of unconventional T cells such as invariant Natural Killer T cells (44), MR1-specific MAIT cells (45) and Gamma-Delta (γδ) T cells (46, 47). These immune cells have been shown to play important roles in suppressing tumor progression by demonstrating both direct tumor cell killing and suppression of pro-tumor immune cells (48). In addition, the expression of *ZBTB16* has been shown in other cancers like Breast Cancer to directly induce G2/M phase cell cycle arrest, apoptosis, and inhibition of migration and invasion in these cancer cells (49). The second most upregulated gene within the Positive Regulation of T Cell Differentiation term was *EGR3* (**Figure 8B**). *EGR3*, in addition to its role in regulation of immune cell activation, was recently determined to be a critical metastasis suppressor in other cancers like prostate cancer (50). These results suggest that C/T in HGSOC patients with delayed recurrence does not only induce cancer cell death but also modulates the tumor immune microenvironment and maintains an anti-tumor phenotype.

Based on the pattern of DEGs, iPathwayGuide also has the functionality to predict genes that may be activated (or inhibited) upstream of the observed differential gene expression signature (26). In patients with Late Recurrence, the upstream regulators predicted as activated post-C/T compared to pre-C/T include IL1β (**Figure 8C**) and STAT3 (**Figure 8D**). The target genes of these upstream regulators were all upregulated post-C/T in our cohort of patients with Late Recurrence and included genes important for DNA damage repair and apoptosis as expected, but also those involved in the immune response to cancer (**Figures 8C, D**).

### Delayed recurrence, but not early recurrence of ovarian cancer, is associated with significant reversal of immune tolerance to cancer post-C/T

**Figure 9:**
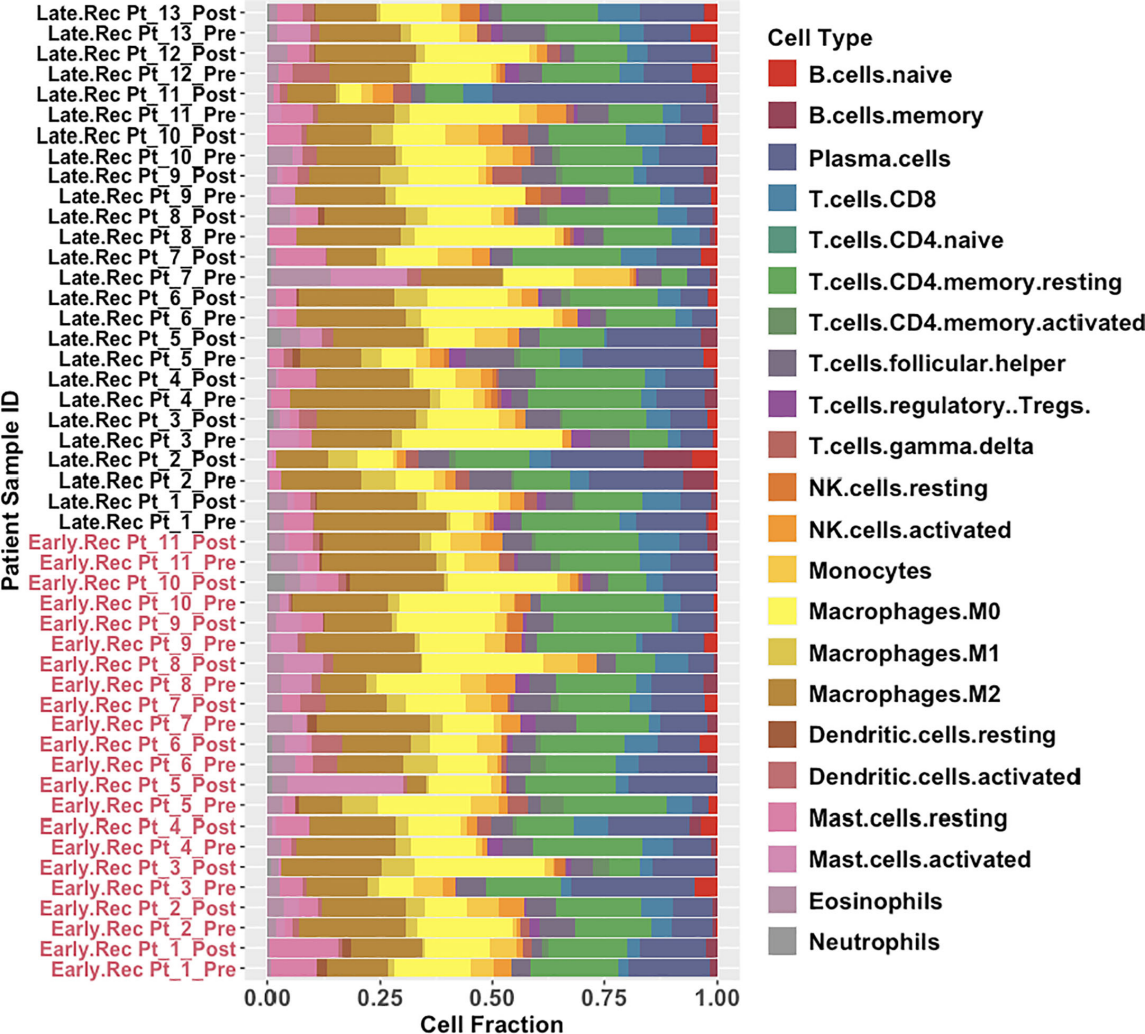
Global results obtained from immune deconvolution of RNA-seq data from HGSOC patients. Stacked bar-plots showing estimated fractions of 22 immune cell types determined using CIBERSORTx’s in-built LM22 signature matrix. Early Recurrence patients are labelled in red while Late Recurrence patients are labelled in black.

Our results so far have shown that chemotherapy in HGSOC patients who would experience delayed recurrence of ovarian cancer, induces significant changes in the expression of genes associated with immunological processes and pathways. These immunological processes and pathways include positive regulation of T cell differentiation and antigen-antibody complex-mediated activation of the complement system. We next performed immune deconvolution of the bulk RNA-seq data using CIBERSORTx to determine if any changes in fractions of pro-tumor and anti-tumor immune cells infiltrating ovarian tumors occurred following C/T. CIBERSORTx estimates immune cell fractions for 22 different immune cell types (51), the combined results of which are shown in **Figure 9**, and then shown separately for relevant cell types subsequently. For pre-C/T comparisons of Late Recurrence versus Early Recurrence patient immune cell fractions, we performed the Wilcoxon Rank Sum Test to determine statistically significant differences. For paired pre-C/T versus post-C/T samples of either the Late Recurrence cohort or the Early Recurrence cohort, we performed the Wilcoxon Signed Rank Test to detect statistically significant changes occurring in immune cell fractions post-C/T. Calculated p-values were corrected for multiple comparison using the Benjamini-Hochberg FDR method.

**Figure 10:**
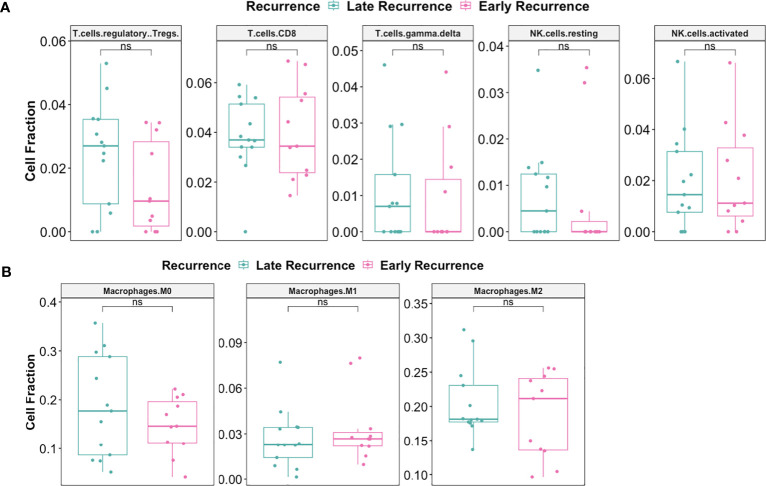
There were no significant differences pre-C/T in immune infiltration of tumors from late recurrence versus early recurrence patients. **(A)** Boxplots comparing pre-C/T estimated cell fractions of T cell and NK cell subtypes between Late Recurrence patients (n=13) and Early Recurrence patients (n=11). P-values shown on plots were calculated from Wilcoxon Rank Sum Test and corrected for multiple comparison using the Benjamini-Hochberg method. **(B)** Boxplots comparing pre-C/T estimated cell fractions of Macrophage subtypes between Late Recurrence patients (n=13) and Early Recurrence patients (n=11). P-values shown on plots were calculated from Wilcoxon Rank Sum Test and corrected for multiple comparison using the Benjamini-Hochberg method. ^ns^ *P* > 0.05; ns, not significant.

Pre-C/T, fractions of tumor infiltrating regulatory T cells (Tregs), CD8+ T cells, γδ T cells, and resting or activated natural killer (NK) cells were not significantly different between patients with Late Recurrence versus Early Recurrence (**Figure 10A**): Tregs (adjusted *P* = 0.75), CD8 T cells (adjusted *P* = 0.95), γδ T cells (adjusted *P* = 0.95), resting NK cells (adjusted *P* = 0.75), and activated NK cells (adjusted *P* = 0.95). In addition, fractions of macrophage subtypes were not significantly different between the two groups pre-C/T (**Figure 10B**): M0 Macrophages (adjusted *P* = 0.61), M1 Macrophages (adjusted *P* = 0.61) and M2 Macrophages (adjusted *P* = 0.61).

**Figure 11:**
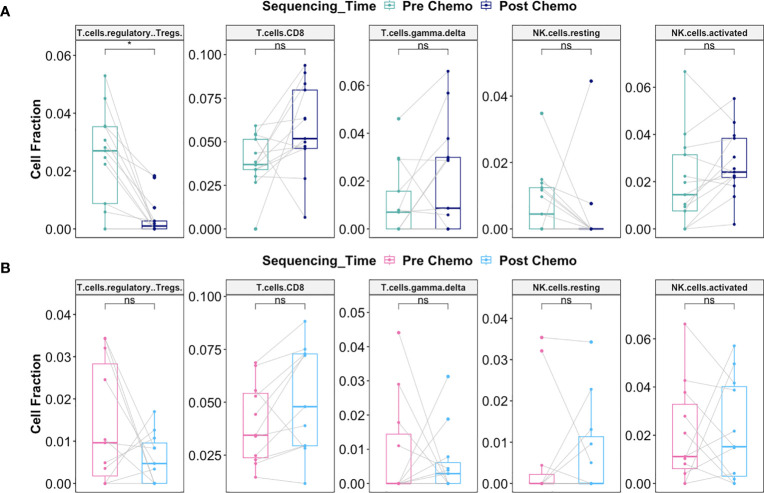
Delayed recurrence of ovarian cancer was associated with significant decrease in T regulatory cells in the tumor microenvironment post-C/T. **(A)** Boxplots comparing estimated cell fractions of T cell and NK cell subtypes between pre-C/T (n=13) versus post-C/T (n=13) samples from Late Recurrence patients. P-values shown on plots are calculated from Wilcoxon Signed Rank Test and corrected for multiple comparison using the Benjamini-Hochberg method. **(B)** Boxplots comparing estimated cell fractions of T cell and NK cell subtypes between pre-C/T (n=11) versus post-C/T (n=11) samples from Early Recurrence patients. P-values shown on plots are calculated from Wilcoxon Signed Rank Test and corrected for multiple comparison using the Benjamini-Hochberg method. ^*^ *P* < 0.05, ^ns^ *P* > 0.05; ns, not significant.

Interestingly, there was significant decrease in the fraction of Regulatory T cells post-C/T in Late Recurrence patients (adjusted *P* = 0.02) (**Figure 11A**) while no statistically significant decrease was observed in Early Recurrence patients (adjusted *P* = 0.55) (**Figure 11B**). CD8^+^-T cell fractions, though not statistically significant, were more likely to increase post-C/T in Late Recurrence patients (adjusted *P* = 0.08) (**Figure 11A**), compared to Early Recurrence patients (adjusted *P* = 0.21) (**Figure 11B**). Both gamma-delta T cells and natural killer cells did not show significant changes post-C/T in either group (**Figures 11A, B**).

**Figure 12:**
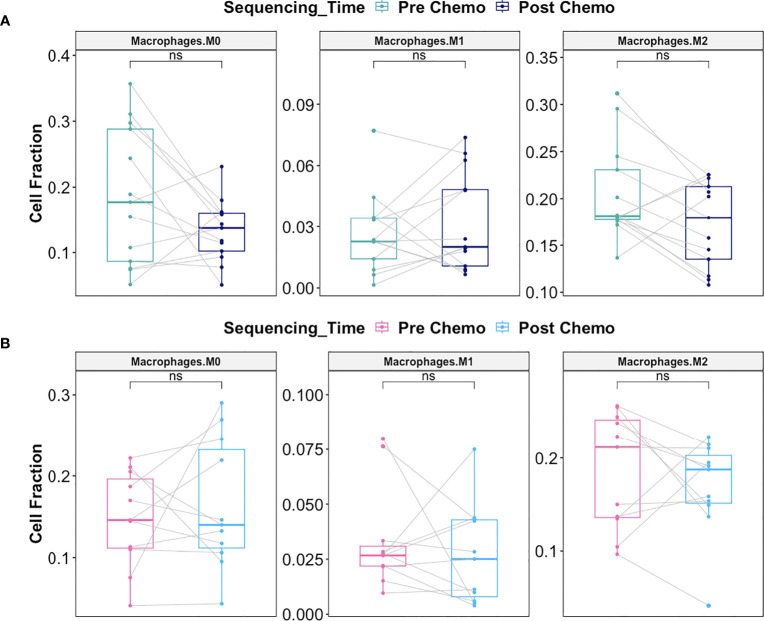
C/T did not induce statistically significant shift in macrophage subtype in Late Recurrence or Early Recurrence patients. **(A)** Boxplots comparing estimated cell fractions of Macrophage subtypes between pre-C/T (n=13) versus post-C/T (n=13) samples from Late Recurrence patients. P-values shown on plots are calculated from Wilcoxon Signed Rank Test and corrected for multiple comparison using the Benjamini-Hochberg method. **(B)** Boxplots comparing estimated cell fractions of Macrophage subtypes between pre-C/T (n=11) versus post-C/T (n=11) samples from Early Recurrence patients. P-values shown on plots are calculated from Wilcoxon Signed Rank Test and corrected for multiple comparison using the Benjamini-Hochberg method. ^ns^ *P* > 0.05; ns, not significant.

Macrophages play a critical role on tumor progression. Consequently, we evaluated whether there was differential response in macrophage infiltration between Late Recurrence versus Early Recurrence patients post-C/T. We observed a decreasing trend in the M2 macrophage fraction post-C/T for most Late Recurrence patients, though this was not statistically significant (adjusted *P* = 0.17) (**Figure 12A**). No such trend was observed for Early Recurrence patients (**Figure 12B**).

Together, our data suggests that, in patients who would show Late Recurrence post-C/T, there is significant reversal of tumor infiltrating immune cell profile from a tolerogenic phenotype towards an anti-tumor profile post-C/T. No such immune profile shift is consistently observed in Early Recurrence patients. The 14 other immune cell types (including neutrophils) compared for Late Recurrence patients (**Supplementary Figure 6**) and Early Recurrence patients (**Supplementary Figure 7**) did not reveal any significant impact of chemotherapy on these populations. Neutrophil-to-Lymphocyte Ratios (NLR) were also calculated by dividing Neutrophil fraction by the sum of all T and B lymphocyte fractions for each patient, and the resulting pre- versus post-chemotherapy NLRs compared. No significant impact of chemotherapy was observed on Neutrophil fraction or Neutrophil-to-Lymphocyte Ratio in either Late Recurrence (**Supplementary Figure 6**) or Early Recurrence patients (**Supplementary Figure 7**).

**Figure 13:**
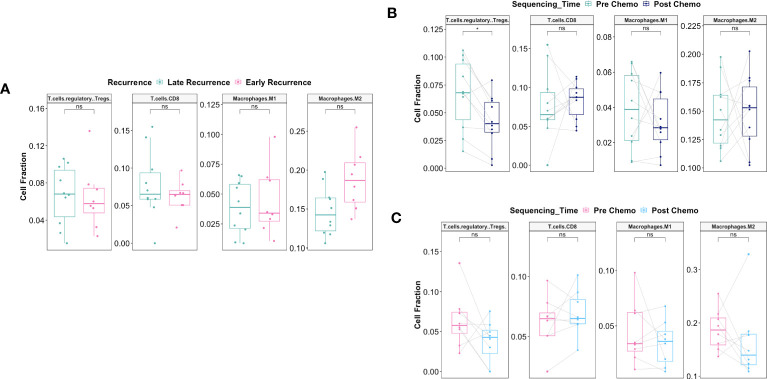
Validation of immune deconvolution results in an independent patient cohort confirms post-C/T decrease of Treg fraction in Late Recurrence patients. **(A)** Boxplots comparing pre-C/T estimated cell fractions of T cell and Macrophage subtypes between Late Recurrence patients (n=10) and Early Recurrence patients (n=8). P-values shown on plots are calculated from Wilcoxon Rank Sum Test and corrected for multiple comparison using the Benjamini-Hochberg method. **(B)** Boxplots comparing estimated cell fractions of T cell and Macrophage subtypes between pre-C/T (n=10) versus post-C/T (n=10) samples from Late Recurrence patients. P-values shown on plots are calculated from Wilcoxon Signed Rank Test and corrected for multiple comparison using the Benjamini-Hochberg method. **(C)** Boxplots comparing estimated cell fractions of T cell and Macrophage subtypes between pre-C/T (n=8) versus post-C/T (n=8) samples from Early Recurrence patients. P-values shown on plots are calculated from Wilcoxon Signed Rank Test and corrected for multiple comparison using the Benjamini-Hochberg method. ^*^ *P* < 0.05, ^ns^ *P* > 0.05.

To validate our findings in an independent cohort of patients, we downloaded paired pre- and post-C/T RNA-seq data for 20 HGSOC patients published by Javellana and colleagues at the University of Chicago (19). One patient whose pre-C/T sample was from pleural effusion (instead of solid tumor) and another patient who showed intermediate response were excluded. Immune cell fractions of the remaining 18 patients – 8 Early Recurrence and 10 Late Recurrence – were estimated using CIBERSORTx. Once more, were observed no statistically significant differences pre-C/T between tumors from Early and Late Recurrence patients (**Figure 13A**). However, Late Recurrence patients showed significant reduction in Tregs post-C/T (adjusted *P* = 0.04) (**Figure 13B**) while no such significant decrease was observed in Early Recurrence patients (**Figure 13C**).

## Discussion

A major unanswered question in ovarian cancer research is why some late-stage HGSOC patients present early recurrence disease after successful initial treatment compared to others who show late recurrence. Finding the answer(s) to this question will have implications for clinical management of HGSOC patients and as such, it is essential to better understand the biology behind the differential response. In the present study we describe a unique immunological signature associated with a positive response to chemotherapy.

Several mechanisms have been proposed to explain differences in patient outcomes. For instance, tumors from poor responders have been proposed to, even before the start of treatment, possess inherent genomic and transcriptomic profiles distinct from tumors from good responders (52). Another hypothesized mechanism is differential ability of cancer cells to suppress uptake (or increase efflux) of chemotherapy drugs, resulting in chemotherapy resistance and potentially facilitating refractoriness to treatment or early cancer recurrence (5).

New evidence suggests that intra-tumoral changes occurring in response to chemotherapy may have a major impact on treatment outcome (19, 53, 54). For instance, using single cell RNA sequencing, a European group found chemotherapy-induced enrichment of stress-related genes in ovarian tumors which was associated with poor prognosis in HGSOC (54). Another group, using bulk RNA sequencing, found chemotherapy-induced upregulation of AP-1 genes particularly in chemo-resistant tumor samples from HGSOC patients (19). These suggest that chemotherapy can induce gene expression changes that impact treatment outcomes.

By studying the transcriptome of HGSOC patients who show late recurrence versus early recurrence patients; and comparing samples before treatment with Carboplatin and Taxol (i.e., pre-C/T) versus post-C/T samples, we generated unique and critical outcomes. In this study, we adopted a stepwise approach to identify the true differences between patients who show Late Recurrence and Early Recurrence. In addition, to determine truly differentially expressed genes, we controlled error rates more strictly by performing quasi-likelihood F-tests with the edgeR package (55) instead of the usual likelihood ratio tests and utilized only FDR-adjusted p-values. Furthermore, we used the more stringent smallest common denominator pruning in iPathwayGuide (26) to identify the most meaningful enriched GO biological processes. We also relied on the more biologically relevant topology-based pathway impact analysis (56) instead of regular overrepresentation analysis or functional class scoring to detect significantly impacted pathways post-C/T.

We found that pre-C/T, there was no statistically significant differences in the transcriptome of ovarian tumors from late recurrence patients versus early recurrence patients. However, in comparing the pre-C/T versus post-C/T transcriptome profiles for each recurrence class, we found that chemotherapy induced significant gene expression changes in tumors from late recurrence patients. On the contrary, the transcriptome of tumors from early recurrence patients did not change significantly following C/T. In patients belonging to the late recurrence cohort, C/T induced downregulation of genes involved in cell proliferation and upregulation of genes involved with apoptosis. This was not surprising since it suggests that chemotherapy effectively induces cancer cell death in patients who would show good response, i.e., delayed (instead of early) recurrence.

Interestingly, we found that C/T also induced significant immunological response in late recurrence patients. Specifically, we demonstrated that when chemotherapy is effective, there is reversal of the immune profile in the tumor microenvironment from a pro-tumor phenotype towards an anti-tumor immune response. To the best of our knowledge, this is the first time that a transcriptomic study in HGSOC patients showed that effective C/T therapy induces an anti-tumor immune response alongside cancer cell death in good responders but not in poor responders.

**Figure 14:**
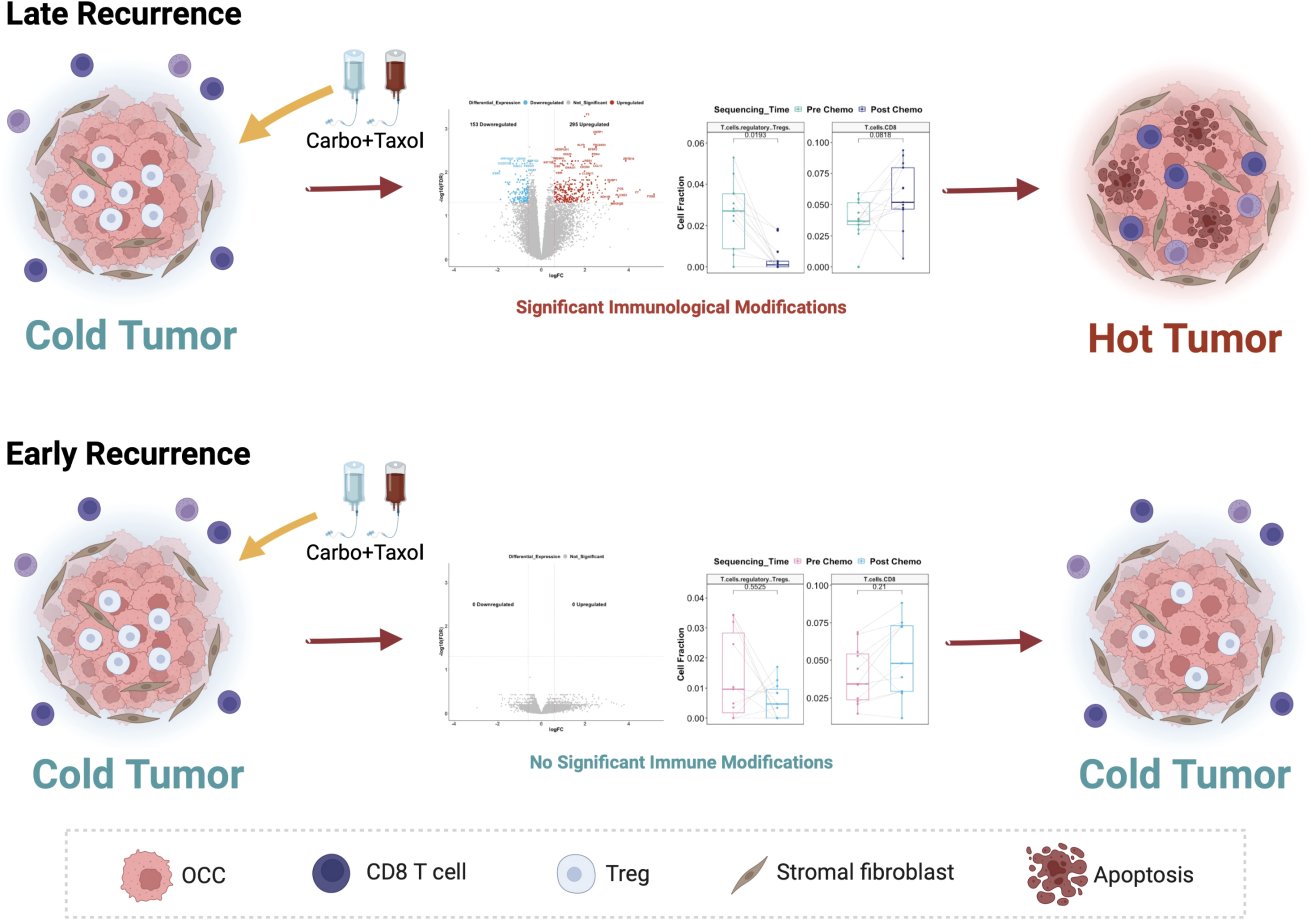
Proposed model of the differential response to Carboplatin plus Taxol in HGSOC patients and its association with different recurrence profiles. In Late Recurrence patients, treatment with Carboplatin and Taxol induces significant gene expression changes and immunological modifications, turning the cold ovarian tumor hot. In Early Recurrence patients, treatment with Carboplatin and Taxol does not induce significant gene expression changes or immunological modifications; hence the tumor remains immunologically cold.

HGSOC has primarily been considered an immunologically “cold tumor”, with Immune Checkpoint Blockage not yet showing significant success (57). “Cold tumors” are cancers with low infiltration of anti-tumor CD8+ T cells and Natural Killer cells, but high presence of immunosuppressive cell types such as regulatory T cells (Tregs), myeloid-derived suppressor cells (MDSCs) and M2 macrophages (58). This is in contrast with immunologically “hot tumors” which have high infiltration of CD8+ T cells and NK cells with low Tregs, MDSCs and M2 macrophages (58). Tregs, in particular, constitute a major cell type which mediate immune tolerance to cancer (i.e., making tumors immunologically cold) and support tumor progression (59). It is known that immunotherapy is effective in immunologically “hot tumors” where it successfully re-invigorates exhausted anti-tumor immune cell infiltrates. This suggests that converting cold tumors into hot tumors could make them more responsive to immunotherapy (58). Therefore, our finding that C/T converts ovarian tumors from being “cold” towards a “hot” phenotype in patients with late recurrence (**Figure 14**) suggests that these patients are more likely to benefit from immunotherapy.

In summary, we report the identification of a unique signature in tumor samples following chemotherapy associated with a long-term response. The intriguing aspect of our findings is the association of immune activation and a better prognosis. Furthermore, our findings also suggest that identifying alternative mechanisms to activate an immune response against cancer in potential “poor responders” at the time of chemotherapy could delay recurrence significantly. Ultimately, this underscores the need for practicing personalized medicine to improve survival for ovarian cancer patients.

## Data availability statement

The datasets presented in this study can be found in online repositories. The names of the repository/repositories and accession number(s) can be found below: Gene Expression Omnibus (GEO) (RRID:SCR_005012) at GSE227100.

## Ethics statement

The studies involving human participants were reviewed and approved by IRB-19-06-1181. The patients/participants provided their written informed consent to participate in this study.

## Author contributions

**NA**: Conceptualization, Investigation, Formal Analysis, Validation, Software, Visualization, Writing. **AA**: Conceptualization, Project Administration, Supervision, Writing. **RA-F**: Conceptualization, Resources. **RG**: Conceptualization. **LC**: Resources. **RT**: Investigation. **HC**: Investigation. **VG**: Formal Analysis. **RM**: Conceptualization. **MA**: Conceptualization, Resources. **JV**: Resources. **CL**: Resources. **DC**: Resources. **SD**: Formal Analysis, Software, Supervision. **TR** Conceptualization, Supervision, Resources, Funding Acquisition. **GM**: Conceptualization, Funding Acquisition, Supervision, Visualization, Writing, Project Administration. All authors contributed to the article and approved the submitted version.

## References

[1] Lheureux S Gourley C Vergote I Oza AM. Epithelial ovarian cancer. Lancet (2019) 393(10177):1240–53. doi: 10.1016/S0140-6736(18)32552-2 30910306

[2] Pignata S Cecere SC Du Bois A Harter P Heitz F. Treatment of recurrent ovarian cancer. Ann Oncol (2017) 28(suppl_8):viii51–6. doi: 10.1093/annonc/mdx441 29232464

[3] Matz M Coleman MP Carreira H Salmeron D Chirlaque MD Allemani C. Worldwide comparison of ovarian cancer survival: histological group and stage at diagnosis (CONCORD-2). Gynecol Oncol (2017) 144(2):396–404. doi: 10.1016/j.ygyno.2016.11.019 27919574 PMC6195190

[4] Freimund AE Beach JA Christie EL Bowtell DDL. Mechanisms of drug resistance in high-grade serous ovarian cancer. Hematol Oncol Clin North Am (2018) 32(6):983–96. doi: 10.1016/j.hoc.2018.07.007 30390769

[5] Ortiz M Wabel E Mitchell K Horibata S. Mechanisms of chemotherapy resistance in ovarian cancer. Cancer Drug Resistance (2022) 5(2):304–16. doi: 10.20517/cdr.2021.147 35800369 PMC9255249

[6] Pogge von Strandmann E Reinartz S Wager U Muller R. Tumor-host cell interactions in ovarian cancer: pathways to therapy failure. Trends Cancer (2017) 3(2):137–48. doi: 10.1016/j.trecan.2016.12.005 28718444

[7] Yang Y Yang Y Yang J Zhao X Wei X. Tumor microenvironment in ovarian cancer: function and therapeutic strategy. Front Cell Dev Biol (2020) 8:758. doi: 10.3389/fcell.2020.00758 32850861 PMC7431690

[8] Chehade H Tedja R Ramos H Bawa TS Adzibolosu N Gogoi R. Regulatory role of the adipose microenvironment on ovarian cancer progression. Cancers (2022) 14(9):2267. doi: 10.3390/cancers14092267 35565396 PMC9101128

[9] Zhang L Conejo-Garcia JR Katsaros D Gimotty PA Massobrio M Regnani G. Intratumoral T cells, recurrence, and survival in epithelial ovarian cancer. N Engl J Med (2003) 348(3):203–13. doi: 10.1056/NEJMoa020177 12529460

[10] Bosmuller HC Wagner P Peper JK Schuster H Pham DL Greif K. Combined immunoscore of CD103 and CD3 identifies long-term survivors in high-grade serous ovarian cancer. Int J Gynecol Cancer (2016) 26(4):671–9. doi: 10.1097/IGC.0000000000000672 26905331

[11] Konstantinopoulos PA Waggoner S Vidal GA Mita M Moroney JW Holloway R. Single-arm phases 1 and 2 trial of niraparib in combination with pembrolizumab in patients with recurrent platinum-resistant ovarian carcinoma. JAMA Oncol (2019) 5(8):1141–9. doi: 10.1001/jamaoncol.2019.1048 PMC6567832 31194228

[12] Pinto MP Balmaceda C Bravo ML Kato S Villarroel A Owen GI. Patient inflammatory status and CD4+/CD8+ intraepithelial tumor lymphocyte infiltration are predictors of outcomes in high-grade serous ovarian cancer. Gynecol Oncol (2018) 151(1):10–7. doi: 10.1016/j.ygyno.2018.07.025 30078505

[13] Sato E Olson SH Ahn J Bundy B Nishikawa H Qian F. Intraepithelial CD8+ tumor-infiltrating lymphocytes and a high CD8+/regulatory T cell ratio are associated with favorable prognosis in ovarian cancer. Proc Natl Acad Sci U.S.A. (2005) 102(51):18538–43. doi: 10.1073/pnas.0509182102 PMC1311741 16344461

[14] Wang Q Lou W Di W Wu X. Prognostic value of tumor PD-L1 expression combined with CD8(+) tumor infiltrating lymphocytes in high grade serous ovarian cancer. Int Immunopharmacol (2017) 52:7–14. doi: 10.1016/j.intimp.2017.08.017 28846888

[15] Webb JR Milne K Watson P Deleeuw RJ Nelson BH. Tumor-infiltrating lymphocytes expressing the tissue resident memory marker CD103 are associated with increased survival in high-grade serous ovarian cancer. Clin Cancer Res (2014) 20(2):434–44. doi: 10.1158/1078-0432.CCR-13-1877 24190978

[16] Disis ML Taylor MH Kelly K Beck JT Gordon M Moore KM. Efficacy and safety of avelumab for patients with recurrent or refractory ovarian cancer: phase 1b results from the JAVELIN solid tumor trial. JAMA Oncol (2019) 5(3):393–401. doi: 10.1001/jamaoncol.2018.6258 30676622 PMC6439837

[17] Hamanishi J Mandai M Ikeda T Minami M Kawaguchi A Murayama T. Safety and antitumor activity of anti-PD-1 antibody, nivolumab, in patients with platinum-resistant ovarian cancer. J Clin Oncol (2015) 33(34):4015–22. doi: 10.1200/JCO.2015.62.3397 26351349

[18] Matulonis UA Shapira-Frommer R Santin AD Lisyanskaya AS Pignata S Vergote I. Antitumor activity and safety of pembrolizumab in patients with advanced recurrent ovarian cancer: results from the phase II KEYNOTE-100 study. Ann Oncol (2019) 30(7):1080–7. doi: 10.1093/annonc/mdz135 31046082

[19] Javellana M Eckert MA Heide J Zawieracz K Weigert M Ashley S. Neoadjuvant chemotherapy induces genomic and transcriptomic changes in ovarian cancer. Cancer Res (2022) 82(1):169–76. doi: 10.1158/0008-5472.CAN-21-1467 PMC8936832 34737212

[20] Garces de Los Fayos Alonso I Liang HC Turner SD Lagger S Merkel O Kenner L. The role of activator protein-1 (AP-1) family members in CD30-positive lymphomas. Cancers (2018) 10(4):93. doi: 10.3390/cancers10040093 29597249 PMC5923348

[21] Calderaro J Meunier L Nguyen CT Boubaya M Caruso S Luciani A. ESM1 as a marker of macrotrabecular-massive hepatocellular carcinoma. Clin Cancer Res (2019) 25(19):5859–65. doi: 10.1158/1078-0432.CCR-19-0859 31358545

[22] Gu X Zhang J Shi Y Shen H Li Y Chen Y. ESM1/HIF−1alpha pathway modulates chronic intermittent hypoxia−induced non−small−cell lung cancer proliferation, stemness and epithelial−mesenchymal transition. Oncol Rep (2021) 45(3):1226–34. doi: 10.3892/or.2020.7913 33650648

[23] Li J Yang D Zhang C Wei S Zhao R Dai S. ESM1 is a promising therapeutic target and prognostic indicator for esophageal Carcinogenesis/Esophageal squamous cell carcinoma. BioMed Res Int (2022) 2022:5328192. doi: 10.1155/2022/5328192 35937390 PMC9348936

[24] Pan KF Lee WJ Chou CC Yang YC Chang YC Chien MH. Direct interaction of beta-catenin with nuclear ESM1 supports stemness of metastatic prostate cancer. EMBO J (2021) 40(4):e105450. doi: 10.15252/embj.2020105450 33347625 PMC7883293

[25] Jantzen SG Sutherland BJ Minkley DR Koop BF. GO trimming: systematically reducing redundancy in large gene ontology datasets. BMC Res Notes (2011) 4:267. doi: 10.1186/1756-0500-4-267 21798041 PMC3160396

[26] Ahsan S Draghici S. Identifying significantly impacted pathways and putative mechanisms with iPathwayGuide. Curr Protoc Bioinf (2017) 57:7 15 1–7 15 30. doi: 10.1002/cpbi.24 28654712

[27] Alexa A Rahnenfuhrer J Lengauer T. Improved scoring of functional groups from gene expression data by decorrelating GO graph structure. Bioinformatics (2006) 22(13):1600–7. doi: 10.1093/bioinformatics/btl140 16606683

[28] Bocci G Di Paolo A Danesi R. The pharmacological bases of the antiangiogenic activity of paclitaxel. Angiogenesis (2013) 16(3):481–92. doi: 10.1007/s10456-013-9334-0 PMC3682088 23389639

[29] Maman S Sagi-Assif O Yuan W Ginat R Meshel T Zubrilov I. The beta subunit of hemoglobin (HBB2/HBB) suppresses neuroblastoma growth and metastasis. Cancer Res (2017) 77(1):14–26. doi: 10.1158/0008-5472.CAN-15-2929 27793844

[30] Ponzetti M Capulli M Angelucci A Ventura L Monache SD Mercurio C. Non-conventional role of haemoglobin beta in breast malignancy. Br J Cancer (2017) 117(7):994–1006. doi: 10.1038/bjc.2017.247 28772282 PMC5625664

[31] Woong-Shick A Sung-Pil P Su-Mi B Joon-Mo L Sung-Eun N Gye-Hyun N. Identification of hemoglobin-alpha and -beta subunits as potential serum biomarkers for the diagnosis and prognosis of ovarian cancer. Cancer Sci (2005) 96(3):197–201. doi: 10.1111/j.1349-7006.2005.00029.x 15771624 PMC11158023

[32] Su T Zhang P Zhao F Zhang S. A novel immune-related prognostic signature in epithelial ovarian carcinoma. Aging (2021) 13(7):10289–311. doi: 10.18632/aging.202792 PMC8064207 33819196

[33] Gibbons DL Abeler-Dorner L Raine T Hwang IY Jandke A Wencker M. Cutting edge: regulator of G protein signaling-1 selectively regulates gut T cell trafficking and colitic potential. J Immunol (2011) 187(5):2067–71. doi: 10.4049/jimmunol.1100833 PMC3166702 21795595

[34] Kumar BV Ma W Miron M Granot T Guyer RS Carpenter DJ. Human tissue-resident memory T cells are defined by core transcriptional and functional signatures in lymphoid and mucosal sites. Cell Rep (2017) 20(12):2921–34. doi: 10.1016/j.celrep.2017.08.078 PMC5646692 28930685

[35] Ye T Zhang X Dong Y Liu J Zhang W Wu F. Chemokine CCL17 affects local immune infiltration characteristics and early prognosis value of lung adenocarcinoma. Front Cell Dev Biol (2022) 10:816927. doi: 10.3389/fcell.2022.816927 35321241 PMC8936957

[36] Marshall LA Marubayashi S Jorapur A Jacobson S Zibinsky M Robles O. Tumors establish resistance to immunotherapy by regulating t(reg) recruitment via CCR4. J Immunother Cancer (2020) 8(2):e000764. doi: 10.1136/jitc-2020-000764 33243932 PMC7692993

[37] Xia ZN Wang XY Cai LC Jian WG Zhang C. IGLL5 is correlated with tumor-infiltrating immune cells in clear cell renal cell carcinoma. FEBS Open Bio (2021) 11(3):898–910. doi: 10.1002/2211-5463.13085 PMC7931224 33449444

[38] Luger D Yang YA Raviv A Weinberg D Banerjee S Lee MJ. Expression of the b-cell receptor component CD79a on immature myeloid cells contributes to their tumor promoting effects. PloS One (2013) 8(10):e76115. doi: 10.1371/journal.pone.0076115 24146823 PMC3797715

[39] Huse K Bai B Hilden VI Bollum LK Vatsveen TK Munthe LA. Mechanism of CD79A and CD79B support for IgM+ b cell fitness through b cell receptor surface expression. J Immunol (2022) 209(10):2042–53. doi: 10.4049/jimmunol.2200144 PMC9643646 36426942

[40] Hosoya N Miyagawa K. Synaptonemal complex proteins modulate the level of genome integrity in cancers. Cancer Sci (2021) 112(3):989–96. doi: 10.1111/cas.14791 PMC7935773 33382503

[41] Takemoto K Imai Y Saito K Kawasaki T Carlton PM Ishiguro KI. Sycp2 is essential for synaptonemal complex assembly, early meiotic recombination and homologous pairing in zebrafish spermatocytes. PloS Genet (2020) 16(2):e1008640. doi: 10.1371/journal.pgen.1008640 32092049 PMC7062287

[42] Yi F Wang Z Liu J Zhang Y Wang Z Xu H. Structural maintenance of chromosomes protein 1: role in genome stability and tumorigenesis. Int J Biol Sci (2017) 13(8):1092–9. doi: 10.7150/ijbs.21206 PMC5599913 28924389

[43] Almitairi JOM Venkatraman Girija U Furze CM Simpson-Gray X Badakshi F Marshall JE. Structure of the C1r-C1s interaction of the C1 complex of complement activation. Proc Natl Acad Sci U.S.A. (2018) 115(4):768–73. doi: 10.1073/pnas.1718709115 PMC5789954 29311313

[44] Park JY DiPalma DT Kwon J Fink J Park JH. Quantitative difference in PLZF protein expression determines iNKT lineage fate and controls innate CD8 T cell generation. Cell Rep (2019) 27(9):2548–2557 e4. doi: 10.1016/j.celrep.2019.05.012 31141681 PMC8274958

[45] Savage AK Constantinides MG Han J Picard D Martin E Li B. The transcription factor PLZF directs the effector program of the NKT cell lineage. Immunity (2008) 29(3):391–403. doi: 10.1016/j.immuni.2008.07.011 18703361 PMC2613001

[46] Cheng ZY He TT Gao XM Zhao Y Wang J. ZBTB transcription factors: key regulators of the development, differentiation and effector function of T cells. Front Immunol (2021) 12:713294. doi: 10.3389/fimmu.2021.713294 34349770 PMC8326903

[47] Lu Y Cao X Zhang X Kovalovsky D. PLZF controls the development of fetal-derived IL-17+Vgamma6+ gammadelta T cells. J Immunol (2015) 195(9):4273–81. doi: 10.4049/jimmunol.1500939 PMC4610873 26408661

[48] Li YR Wilson M Yang L. Target tumor microenvironment by innate T cells. Front Immunol (2022) 13:999549. doi: 10.3389/fimmu.2022.999549 36275727 PMC9582148

[49] He J Wu M Xiong L Gong Y Yu R Peng W. BTB/POZ zinc finger protein ZBTB16 inhibits breast cancer proliferation and metastasis through upregulating ZBTB28 and antagonizing BCL6/ZBTB27. Clin Epigenet (2020) 12(1):82. doi: 10.1186/s13148-020-00867-9 PMC7285556 32517789

[50] Shin SH Kim I Lee JE Lee M Park JW. Loss of EGR3 is an independent risk factor for metastatic progression in prostate cancer. Oncogene (2020) 39(36):5839–54. doi: 10.1038/s41388-020-01418-5 32796959

[51] Newman AM Steen CB Liu CL Gentles AJ Chaudhuri AA Scherer F. Determining cell type abundance and expression from bulk tissues with digital cytometry. Nat Biotechnol (2019) 37(7):773–82. doi: 10.1038/s41587-019-0114-2 PMC6610714 31061481

[52] Hollis RL Gourley C. Genetic and molecular changes in ovarian cancer. Cancer Biol Med (2016) 13(2):236–47. doi: 10.20892/j.issn.2095-3941.2016.0024 PMC4944549 27458531

[53] Bohm S Montfort A Pearce OM Topping J Chakravarty P Everitt GL. Neoadjuvant chemotherapy modulates the immune microenvironment in metastases of tubo-ovarian high-grade serous carcinoma. Clin Cancer Res (2016) 22(12):3025–36. doi: 10.1158/1078-0432.CCR-15-2657 27306793

[54] Zhang K Erkan EP Jamalzadeh S Dai J Andersson N Kaipio K. Longitudinal single-cell RNA-seq analysis reveals stress-promoted chemoresistance in metastatic ovarian cancer. Sci Adv (2022) 8(8):eabm1831. doi: 10.1126/sciadv.abm1831 35196078 PMC8865800

[55] Chen Y Lun AT Smyth GK. From reads to genes to pathways: differential expression analysis of RNA-seq experiments using rsubread and the edgeR quasi-likelihood pipeline. F1000Res (2016) 5:1438. doi: 10.12688/f1000research.8987.2 27508061 PMC4934518

[56] Draghici S Khatri P Tarca AL Amin K Done A Voichita C. A systems biology approach for pathway level analysis. Genome Res (2007) 17(10):1537–45. doi: 10.1101/gr.6202607 PMC1987343 17785539

[57] Wu JWY Dand S Doig L Papenfuss AT Scott CL Ho G. T-Cell receptor therapy in the treatment of ovarian cancer: a mini review. Front Immunol (2021) 12:672502. doi: 10.3389/fimmu.2021.672502 33927729 PMC8076633

[58] Zhang J Huang D Saw PE Song E. Turning cold tumors hot: from molecular mechanisms to clinical applications. Trends Immunol (2022) 43(7):523–45. doi: 10.1016/j.it.2022.04.010 35624021

[59] Scott EN Gocher AM Workman CJ Vignali DAA. Regulatory T cells: barriers of immune infiltration into the tumor microenvironment. Front Immunol (2021) 12:702726. doi: 10.3389/fimmu.2021.702726 34177968 PMC8222776

