# Supplementary material for: Immunological modifications following chemotherapy are associated with delayed recurrence of ovarian cancer
Source: Front Immunol. 2023 Jun 26;14:1204148. doi: 10.3389/fimmu.2023.1204148 (PMC10331425; doi:10.3389/fimmu.2023.1204148)

Supplementary Figure Legends

Supplementary Figure 1: Principal Component Analysis of RNA-sequence data from all tumor samples. The greatest separation of samples is seen to be due to sampling time in relation to treatment (Pre-chemotherapy versus Post-chemotherapy) and not time of recurrence (Early Recurrence versus Late Recurrence).

Supplementary Figure 2: t-Distributed Stochastic Neighbor Embedding (t-SNE) visualization of tumor transcriptomic data. Samples are seen to cluster based on sampling time (Pre-chemotherapy versus Post-chemotherapy) and not time of recurrence (Early Recurrence versus Late Recurrence).

Supplementary Figure 3: Visualization of tumor sample RNA-sequence data using Uniform Manifold Approximation and Projection (UMAP). Samples are seen to cluster based on sampling time (Pre-chemotherapy versus Post-chemotherapy) and not time of recurrence (Early Recurrence versus Late Recurrence).

Supplementary Figure 4: KEGG Pathway diagram for Complement and Coagulation Cascade Pathway perturbation analysis using iPathwayGuide software. Within the Complement and Coagulation pathway, each gene's perturbation is calculated by considering the gene's measured log2-foldchange as well as any accumulated perturbation resulting from a differentially expressed upstream gene. Calculated perturbation of genes in the pathway are color-coded according to the color scale shown above the pathway diagram. The most negative perturbation was -2.8 (blue) and the most positive perturbation was 4.5 (red).

Supplementary Figure 5: Representative images of Hematoxylin and Eosin (H&E) stained high grade serous ovarian tumor samples.

Supplementary Figure 6: Paired pre-chemotherapy versus post-chemotherapy boxplots of immune deconvolution results for Late Recurrence patients. The cell types shown here are the remaining 14 immune cell types determined with the CIBERSORTx deconvolution pipeline using the LM22 signature matrix. Included in the last panel is the Neutrophil-to-Lymphocyte ratio for each patient, calculated as (Neutrophil fraction) / (Total Lymphocyte fraction).

Supplementary Figure 7: Paired pre-chemotherapy versus post-chemotherapy boxplots of immune deconvolution results for Early Recurrence patients. The cell types shown here are the remaining 14 immune cell types determined with the CIBERSORTx deconvolution pipeline using the LM22 signature matrix. Included in the last panel is the Neutrophil-to-Lymphocyte ratio for each patient, calculated as (Neutrophil fraction) / (Total Lymphocyte fraction).

Supplementary Figure 1

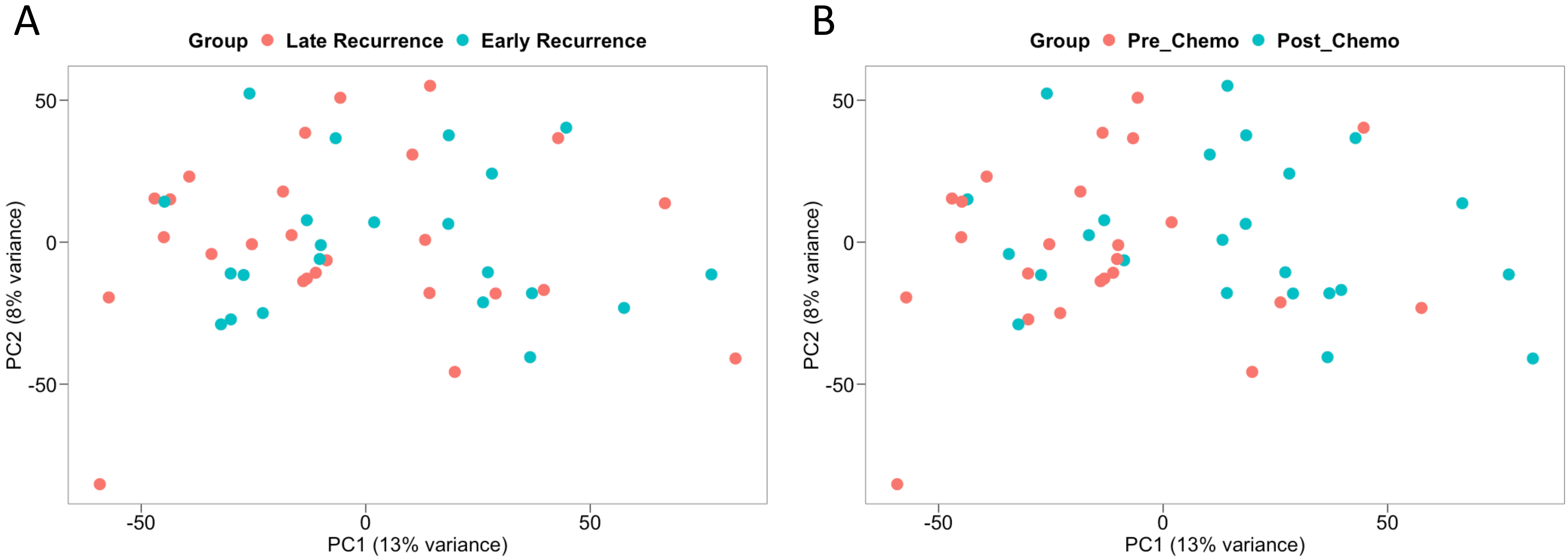

Supplementary Figure 2

A

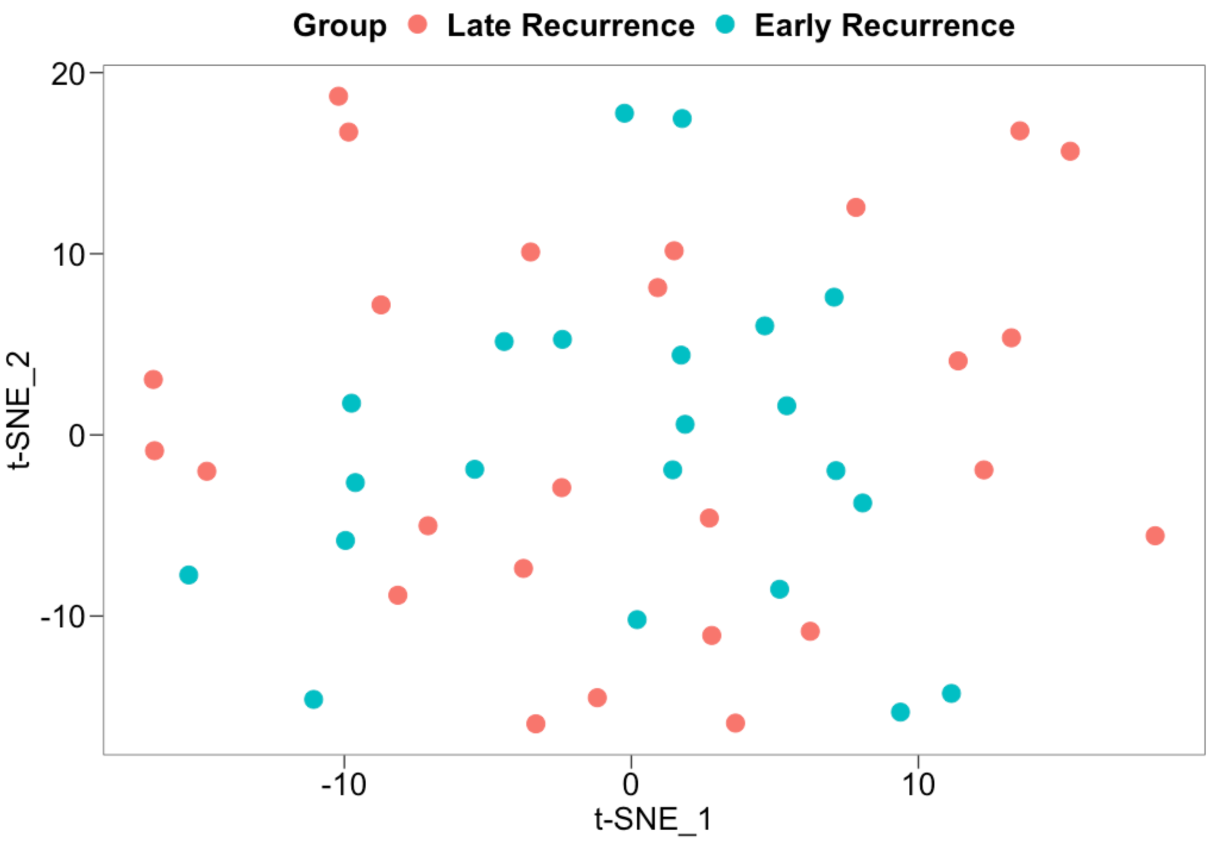

B

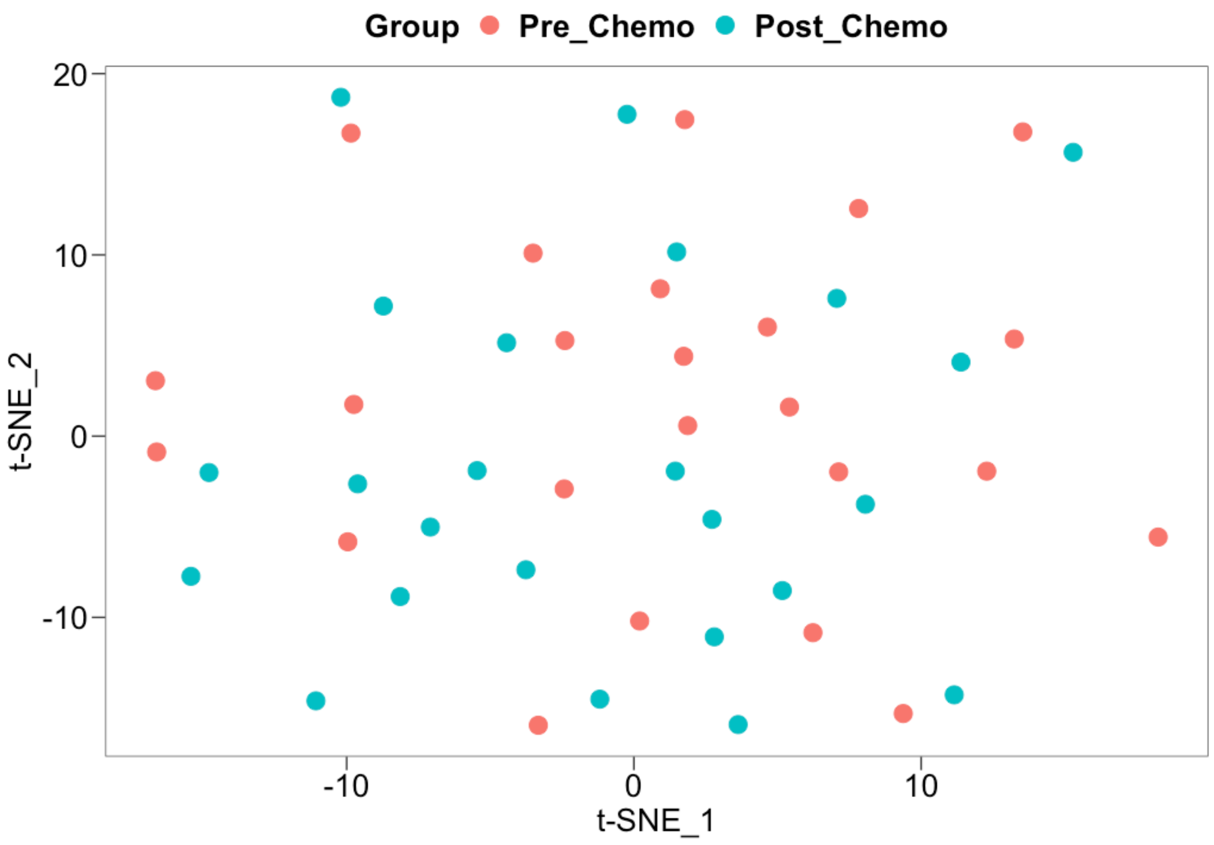

Supplementary Figure 3

A

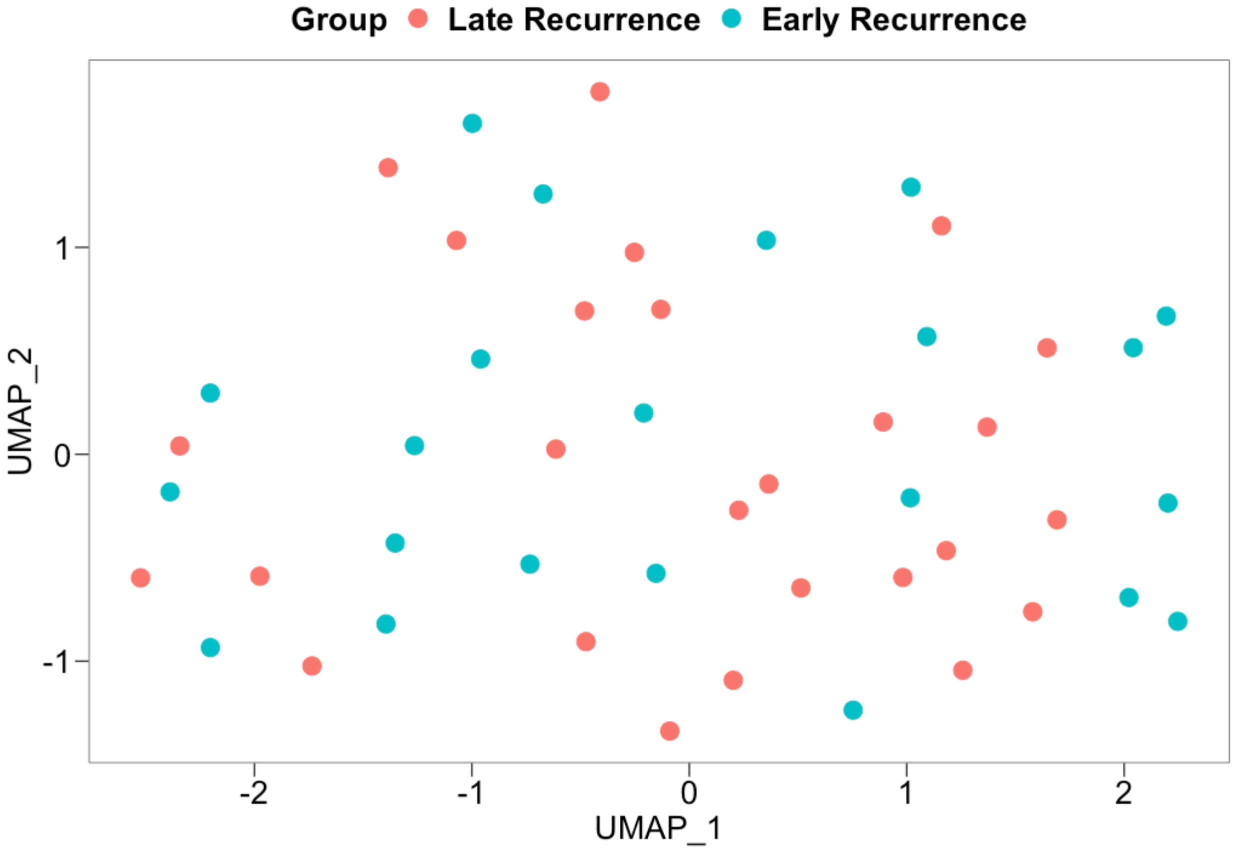

B

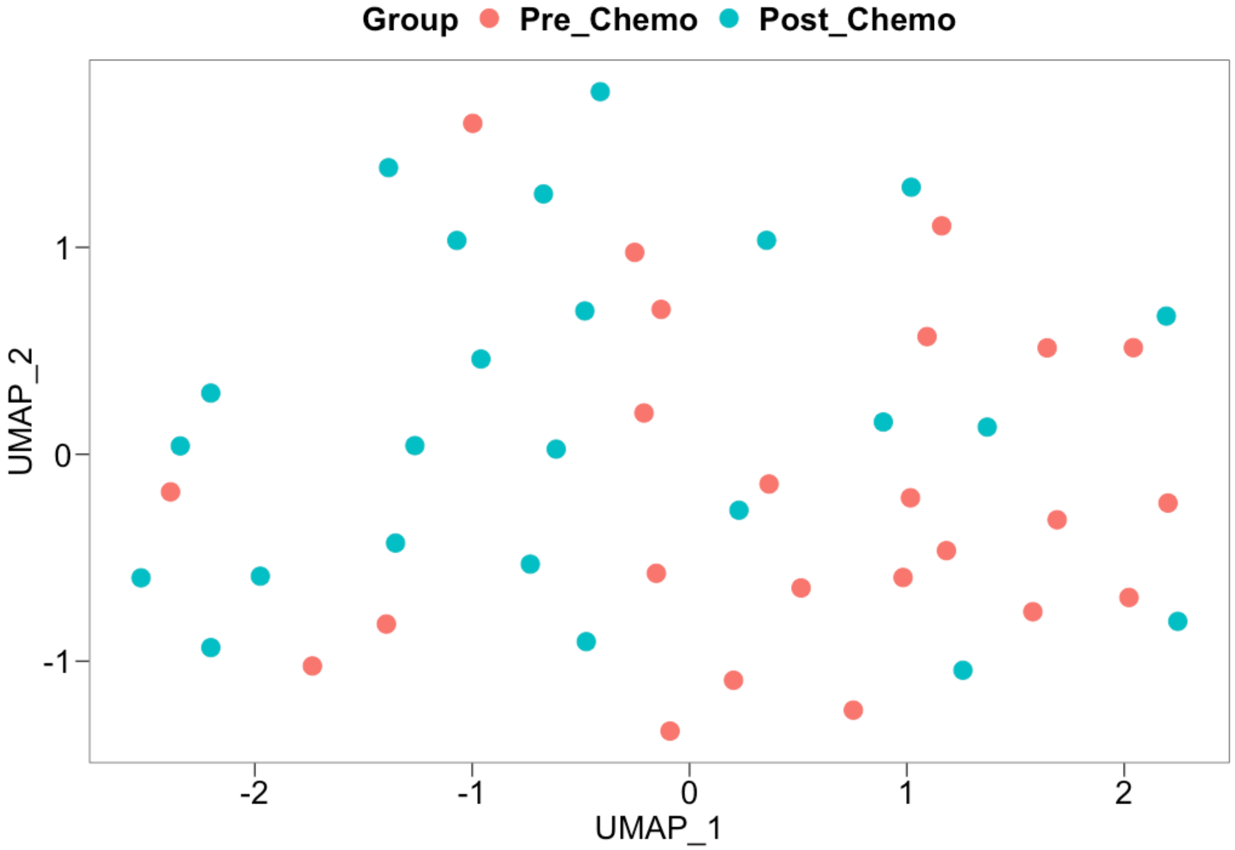

Supplementary Figure 4

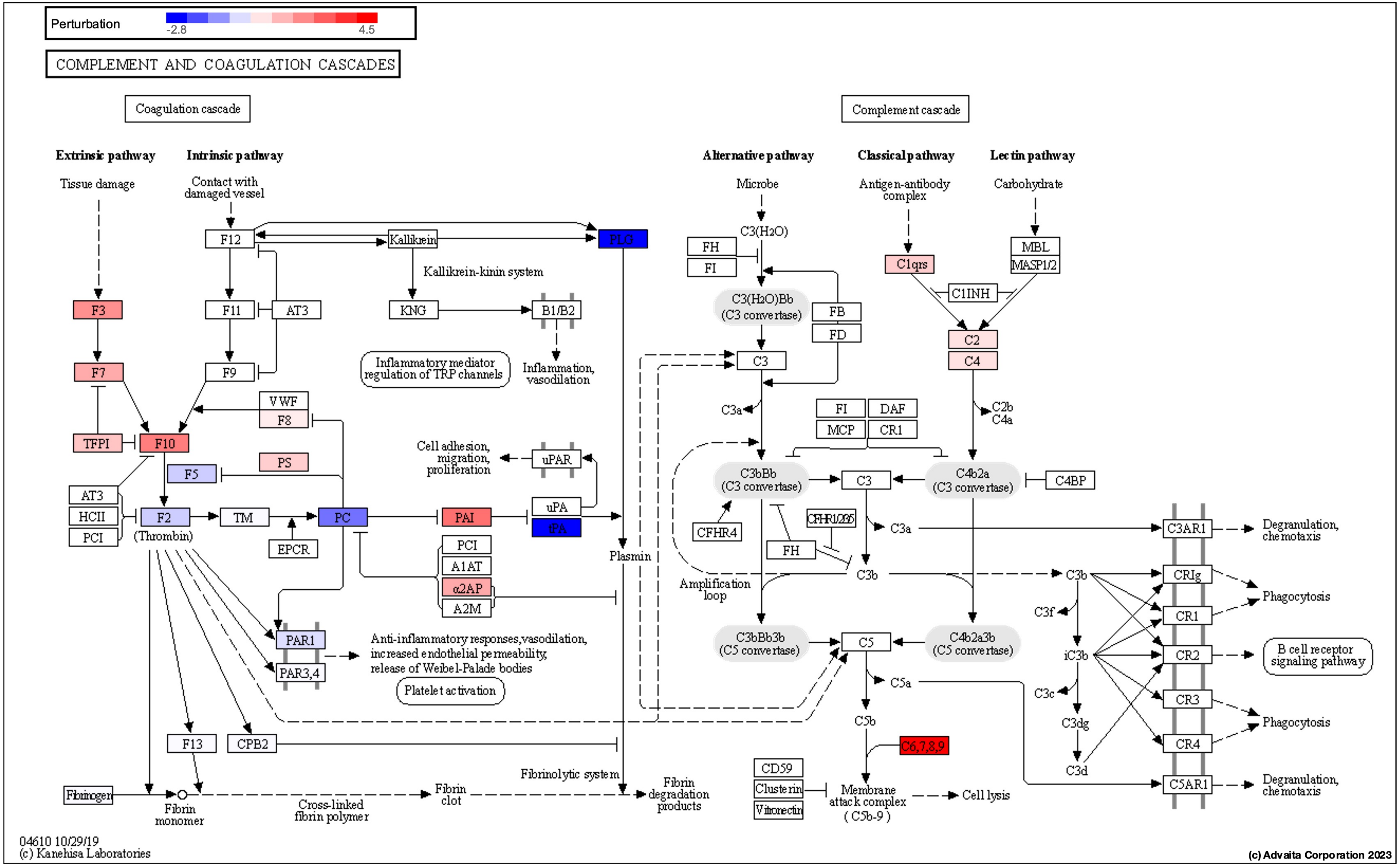

Supplementary Figure 5

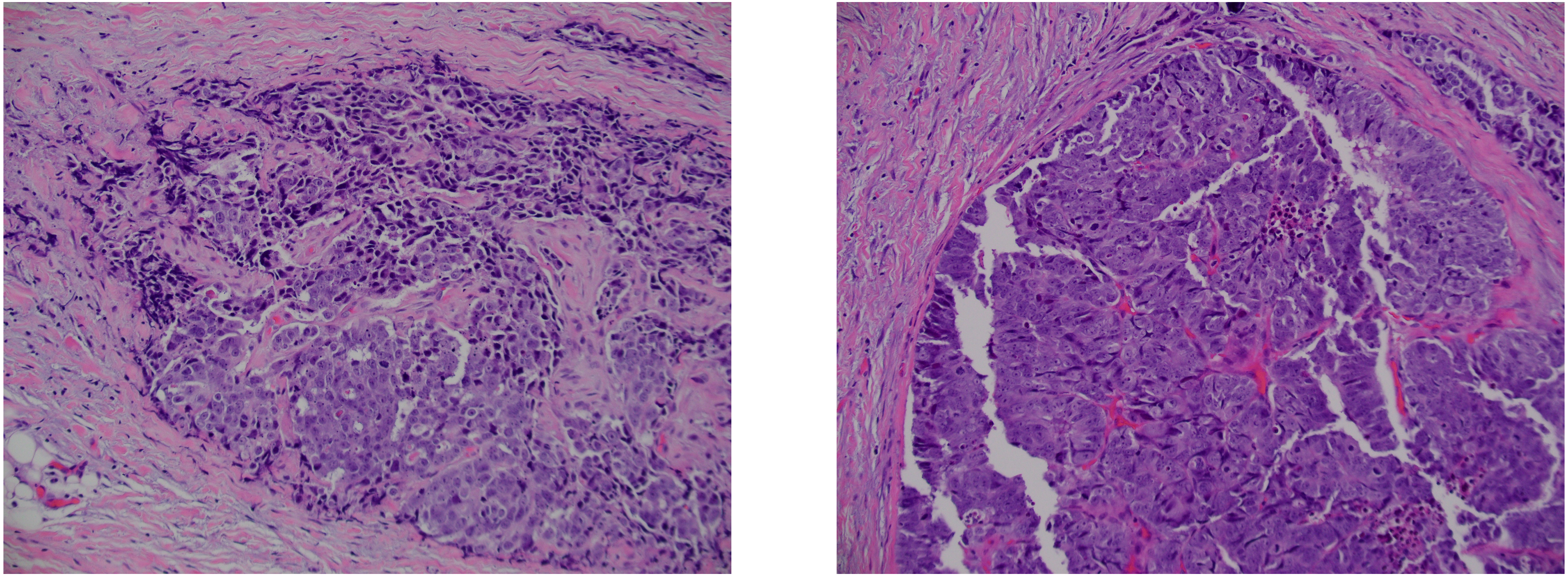

Supplementary Figure 6

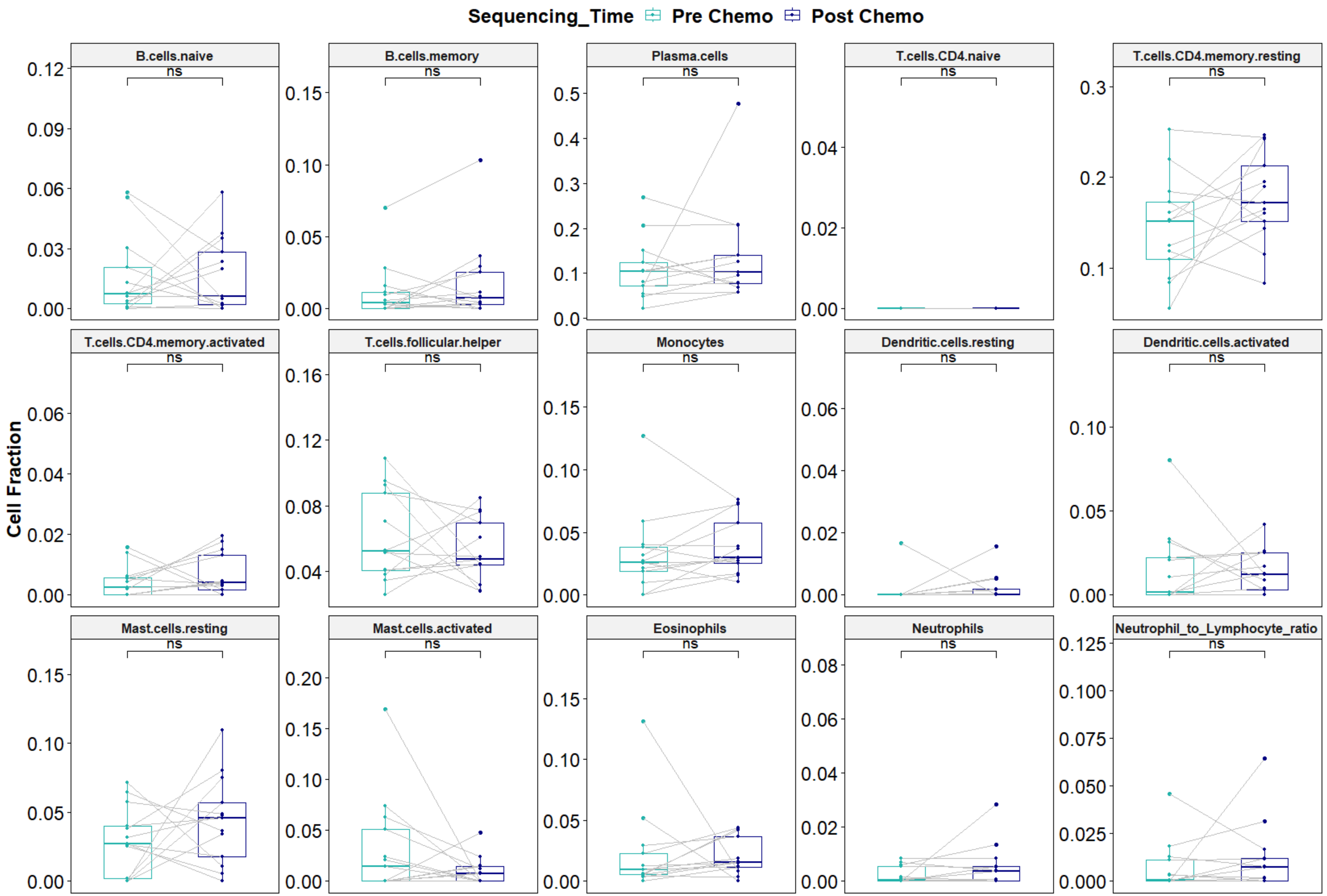

Supplementary Figure 7

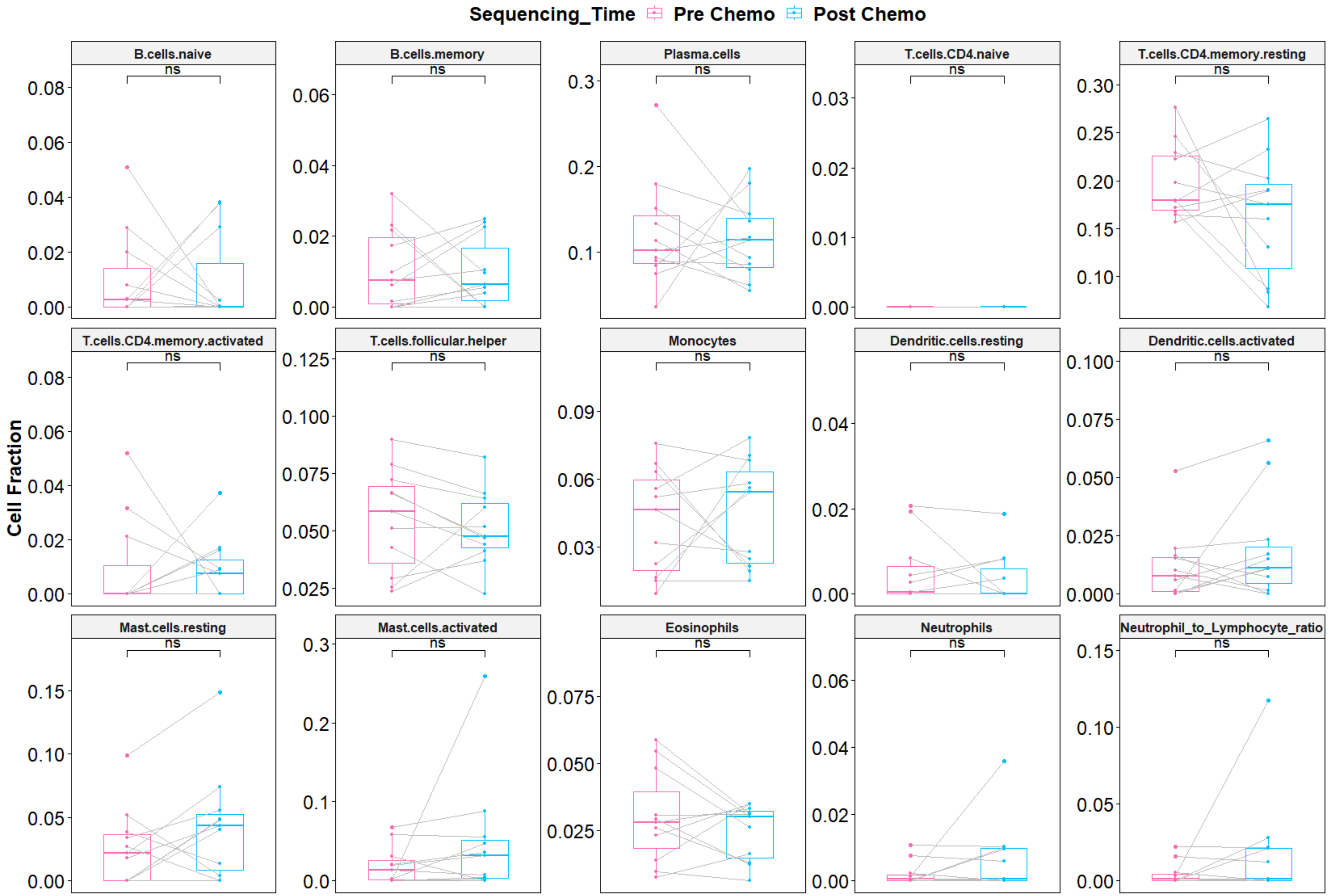

Supplement: Supplementary file 1 [file DataSheet_1.pdf]
